# The Lck inhibitor, AMG-47a, blocks necroptosis and implicates RIPK1 in signalling downstream of MLKL

**DOI:** 10.1038/s41419-022-04740-w

**Published:** 2022-04-01

**Authors:** Annette V. Jacobsen, Catia L. Pierotti, Kym N. Lowes, Amanda E. Au, Ying Zhang, Nima Etemadi, Cheree Fitzgibbon, Wilhelmus J. A. Kersten, André L. Samson, Mark F. van Delft, David C. S. Huang, Hélène Jousset Sabroux, Guillaume Lessene, John Silke, James M. Murphy

**Affiliations:** 1 The Walter and Eliza Hall Institute of Medical Research, Parkville, VIC Australia; 2 Department of Medical Biology, The University of Melbourne, Parkville, VIC Australia; 3 Department of Pharmacology and Therapeutics, The University of Melbourne, Parkville, VIC Australia

**Keywords:** Kinases, Necroptosis

## Abstract

Necroptosis is a form of caspase-independent programmed cell death that arises from disruption of cell membranes by the mixed lineage kinase domain-like (MLKL) pseudokinase after its activation by the upstream kinases, receptor interacting protein kinase (RIPK)-1 and RIPK3, within a complex known as the necrosome. Dysregulated necroptosis has been implicated in numerous inflammatory pathologies. As such, new small molecule necroptosis inhibitors are of great interest, particularly ones that operate downstream of MLKL activation, where the pathway is less well defined. To better understand the mechanisms involved in necroptosis downstream of MLKL activation, and potentially uncover new targets for inhibition, we screened known kinase inhibitors against an activated mouse MLKL mutant, leading us to identify the lymphocyte-specific protein tyrosine kinase (Lck) inhibitor AMG-47a as an inhibitor of necroptosis. We show that AMG-47a interacts with both RIPK1 and RIPK3, that its ability to protect from cell death is dependent on the strength of the necroptotic stimulus, and that it blocks necroptosis most effectively in human cells. Moreover, in human cell lines, we demonstrate that AMG-47a can protect against cell death caused by forced dimerisation of MLKL truncation mutants in the absence of any upstream signalling, validating that it targets a process downstream of MLKL activation. Surprisingly, however, we also found that the cell death driven by activated MLKL in this model was completely dependent on the presence of RIPK1, and to a lesser extent RIPK3, although it was not affected by known inhibitors of these kinases. Together, these results suggest an additional role for RIPK1, or the necrosome, in mediating human necroptosis after MLKL is phosphorylated by RIPK3 and provide further insight into reported differences in the progression of necroptosis between mouse and human cells.

## Introduction

Necroptosis is a caspase-independent form of cell death that involves membrane disruption and the release of cytokines and damage-associated molecular patterns. In mammals, necroptosis appears to have evolved in conjunction with microbial pathogens, as there is evidence for a variety of bacterial and viral proteins that target the necroptosis pathway [1–5]. There is also increasing evidence implicating necroptosis in a range of inflammatory, autoimmune and neurodegenerative diseases (reviewed in [6, 7]) and cancers (reviewed in [8]). For these reasons, there has been much interest in the cell death community in developing inhibitors of necroptosis.

Necroptosis can be activated through engagement of several different extracellular receptors, including death receptors such as tumour necrosis factor receptor 1 (TNFR1), interferon receptors, and Toll-like receptors [9–13]. The pathway can be induced pharmacologically using death ligands such as TNF to engage TNFR1, Smac-mimetics to block the activity of the inhibitor of apoptosis protein (IAP) family of E3 ubiquitin ligases, and caspase inhibitors to prevent the activity of caspases, specifically caspase-8 (CASP8), whose proteolytic activities underlie apoptotic cell death [14]. After engagement of the TNFR1 receptor with TNF, ubiquitylation of receptor interacting protein kinase (RIPK)-1 by cIAPs, which normally prevents cell death, is blocked by the Smac-mimetics. This allows RIPK1 to form a second complex in the cytoplasm that includes CASP8, typically leading to activation of CASP8 and cell death [15–17]. However, in the presence of caspase inhibitors, CASP8 cleavage of RIPK1 is prevented, allowing RIPK1 to associate with RIPK3 through their RIP homotypic interaction motifs (RHIMs) [14, 18–22]. This induces the autophosphorylation and activation of both kinases, allowing RIPK3 to recruit the pseudokinase, mixed lineage kinase domain-like (MLKL), the most downstream known obligate effector of the necroptosis pathway [23–25]. RIPK3 then phosphorylates the activation loop within MLKL’s pseudokinase domain, initiating a chain of events involving a conformational transition, oligomerisation and subsequent translocation of MLKL to cellular membranes, where membrane disruption mediated by MLKL’s N-terminal four-helix bundle (4HB) domain leads to death of the cell [25–36]. Several pathway interactors and modulators of necroptotic signalling have been identified [37–46], however the precise mechanism and regulation of MLKL-mediated plasma membrane disruption is less clear, with endosomal sorting complex proteins, flotillins, phosphatidylinositol phosphates, and ion channels all proposed to have important roles [27–29, 47–52]. Additionally, although there are many similarities between necroptotic signalling in human and mouse cells, key differences have also been shown, particularly in relation to how MLKL is activated by RIPK3 and the nature of the oligomeric complex formed after phosphorylation [30, 31, 33, 53, 54]. This incomplete understanding of the necroptotic signalling pathway poses a challenge to efforts to develop therapeutics.

To unravel some of these outstanding questions, we performed complementary phenotypic screens to identify small molecules that could block necroptosis downstream of MLKL activation, with a view to identifying previously unknown signalling molecules in the pathway. Using this strategy, we previously identified the chaperone, heat shock protein 90 (HSP90), as an MLKL interacting partner [39], a finding supported by two other independent research groups [55, 56]. Here, we describe our identification of the lymphocyte-specific protein tyrosine kinase (Lck) inhibitor, AMG-47a, as an inhibitor of necroptosis via its binding to the two key necroptotic effector kinases, RIPK1 and RIPK3. Surprisingly, while these data implicate RIPK1 as a target of AMG-47a, we also observed that AMG-47a inhibited necroptosis downstream of MLKL activation. Subsequent investigation revealed that death induced by dimerisation of the human MLKL N-terminal domain or overexpression of human RIPK3 is dependent on the presence of RIPK1. Collectively, these data implicate an additional role of RIPK1 in necroptosis downstream of MLKL activation.

## Results

### AMG-47a inhibits necroptosis in a stimulus-dependent manner

**Fig. 1:**
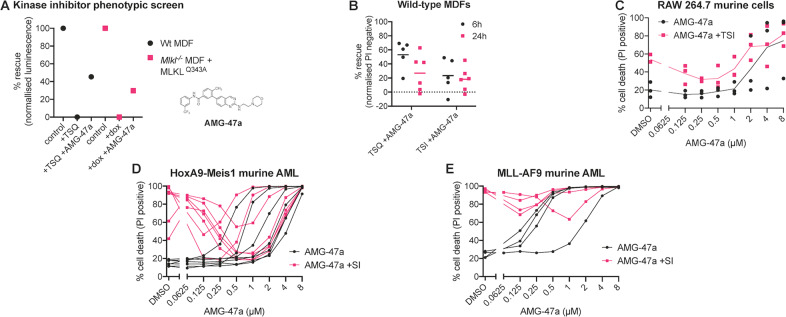
AMG-47a is a novel necroptosis inhibitor identified in mouse cells. **A** AMG-47a was identified in parallel small molecule phenotypic screens to rescue cell death caused by TNF (100 ng/ml), Smac mimetic (0.5 µM) and QVD-OPh (5 µM) (TSQ) in wild-type murine dermal fibroblasts (MDFs), or by induction of MLKL^Q343A^ expression in *Mlkl*^*−/−*^ MDFs using doxycycline (dox; 1 µg/ml) using a CellTiter Glow assay after 24 h treatment. Data are the mean of two technical replicates. **B** Wild-type MDFs were treated with AMG-47a (1 µM) or an equivalent amount of DMSO, then challenged with TSQ or TSI (TNF (100 ng/ml), Smac mimetic (0.5 µM), and caspase inhibitor IDN-6556 (emricasan; 5 µM)) to induce necroptotic cell death or left untreated. After 6 h or 24 h PI uptake was measured using flow cytometry as above. Data were normalised to DMSO treated (0% cell death) and TSQ or TSI treated (100% cell death) and are shown as percentage rescue (see Supplementary Fig. 1A for raw data). **C**–**E** AMG-47a was assessed in murine RAW 264.7 and AML cell lines in 2-fold dilution series for both toxicity (AMG-47a alone), and its ability to protect against induction of necroptosis using TSI or, for murine AML cell lines, Smac mimetic and IDN-6556 alone (SI). Cell death was assessed 24 h after treatment by measuring propidium iodide (PI; 1 µg/ml) uptake using flow cytometry, with a minimum of 5000 cells counted. Data represent three (**C**), seven (**D**) or four (**E**) independent biological replicates, and lines represent the mean (**C**) or individual replicates (**D, E**).

To identify novel inhibitors of necroptosis at the level of, or downstream of, MLKL activation, we used two independent phenotypic screens against a library of known kinase inhibitors, as described previously [39]. In the first screen, wild-type murine dermal fibroblasts (MDFs) were treated with a combination of small molecules known to induce necroptosis (TNF; the Smac-mimetic, Compound A; and the caspase inhibitor QVD-OPh; TSQ). In the second screen, *Mlkl*^*−/−*^ MDFs inducibly-expressing the auto-activating MLKL^Q343A^, were used [25]. From this screen, we found the lymphocyte-specific kinase (Lck) inhibitor, AMG-47a [57], restricted MLKL-dependent cell death in both cell lines (Fig. 1A). To independently validate this, we assessed the ability of AMG-47a to protect against necroptotic cell death induced by either TSQ or TSI (I: the caspase inhibitor IDN-6556) in wild-type MDFs (Fig. 1B). We found that AMG-47a was able to protect against TSQ-mediated cell death and, although there was some variability in the response, this was statistically significant at 6 h (Supplementary Fig. 1A). However, there was less protection against TSI-induced cell death, known to be a stronger inducer of necroptosis [58], even at early time points.

We then assessed whether AMG-47a could protect other murine cell lines shown to die by necroptosis [58, 59]. AMG-47a inhibited TSI-induced necroptosis of RAW cells at a concentration of 0.25–0.5 µM, however toxicity was also observed in cells treated with AMG-47a at doses greater than 1 µM (Fig. 1C). We saw similar toxicity in murine AML cell lines but found there was substantial protection from necroptotic cell death induced by SI in HoxA9-Meis1 AML cells between 125 nM and 1 µM (Fig. 1D, Supplementary Fig. 1B). In contrast, AMG-47a provided little protection against the same necroptotic stimulus in MLL-AF9, MLL-AF9-NRas and MLL-ENL AML cells (Fig. 1E, Supplementary Fig. 1C–E). To understand the variation between the different murine AML cell lines, we examined the expression of necroptosis effectors in HoxA9-Meis1 and MLL-AF9 AML cell lines. Interestingly, both RIPK1 and MLKL were expressed at much lower levels in HoxA9-Meis1 cells (Supplementary Fig.1F), which were the most responsive to AMG-47a inhibition (Fig. 1D).

**Fig. 2:**
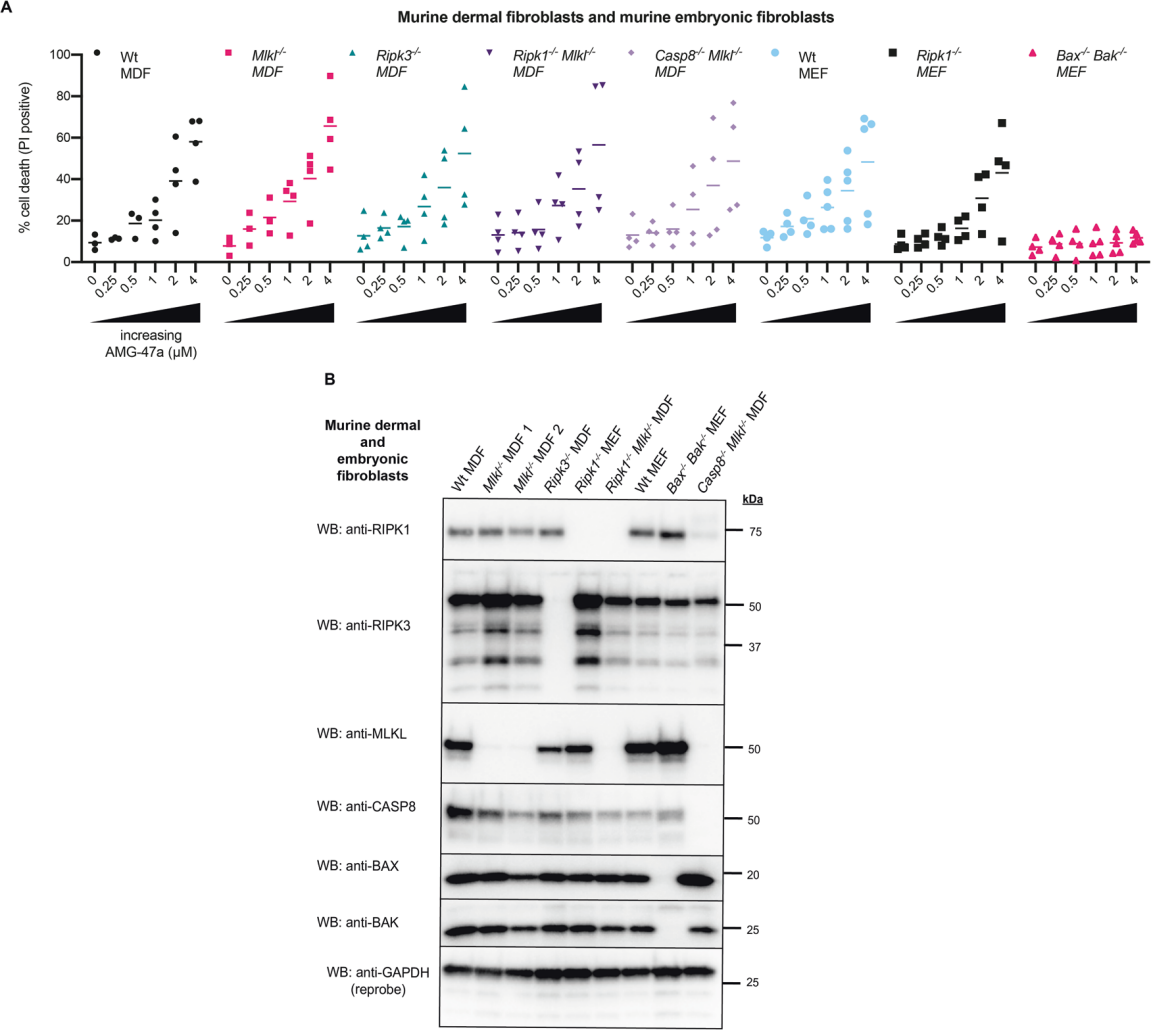
AMG-47a toxicity is Bax/Bak dependent. **A** MDFs and MEFs of various genotypes were treated with increasing doses of AMG-47a, or DMSO alone. After 24 h, cells were harvested and assessed for PI uptake via flow cytometry, with a minimum of 5000 cells counted. Results represent four independent experiments for MDF cell lines and *Ripk1*^*-/-*^ MEFs, and five independent experiments for all other MEF cell lines. **B** Samples of untreated MDFs or MEFs of each genotype used in (**A**) were collected, lysed, and analysed via western blotting to confirm cells of the correct genotype were being used. Data shown are representative of two individual experiments done at different time points over the course of the toxicity experiments.

To address the identified toxicity, we examined whether genetic deletion of key components of different cell death pathways in MDFs or murine embryonic fibroblasts (MEFs) led to protection upon treatment with increasing doses of AMG-47a (Fig. 2A, B). Only *Bax*^*−/−*^*Bak*^*−/−*^ MEFs were completely protected from AMG-47a mediated toxicity (Fig. 2A), suggesting that the compound was initiating apoptosis at higher doses.

**Fig. 3:**
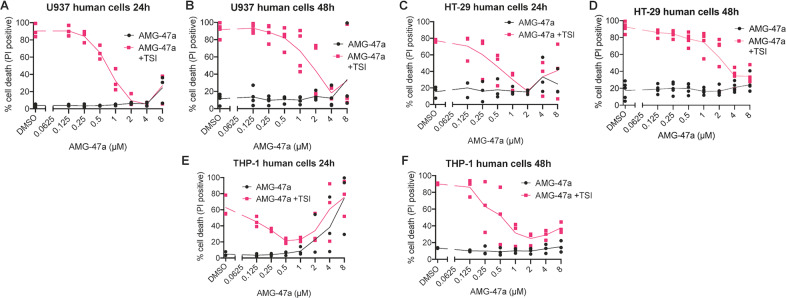
AMG-47a is a strong necroptosis inhibitor in human cell lines. **A**–**F** AMG-47a was assessed in human U937, HT29 and THP-1 cell lines in 2-fold dilution series for both toxicity (AMG-47a alone), and its ability to protect against induction of necroptosis using TSI. Cell death was assessed 24 h (**A**, **C**, **E**) or 48 h (**B**, **D**, **F**) after treatment by measuring propidium iodide (PI; 1 µg/ml) uptake using flow cytometry, with a minimum of 5000 cells counted. Data represent three (**A**, **B**, **E**, **F**) or four (**B**, **D**) independent biological replicates, and lines represent the mean.

We then investigated the ability of AMG-47a to inhibit necroptosis in a range of human cell lines at both 24 and 48 h (Fig. 3A–F, Supplementary Fig. 2A–D). We found that AMG-47a provided robust protection from both TSQ and TSI-induced cell death in U937 histiocytic lymphoma, HT29 colorectal adenocarcinoma and THP-1 acute monocytic leukaemia cells, with IC_50_ values ranging from ~100 nM to ~2.5 µM (Table 1), showing protection comparable to known RIPK1, RIPK3 and MLKL inhibitors (Supplementary Fig. 2E–M). Notably, as in MDFs (Fig. 1B), in U937 and HT29 we found higher doses of AMG-47a were required to block TSI-induced cell death. Also similar to mouse cell lines, we noted some toxicity at higher doses in human cell lines; this was most noticeable in THP-1 cells treated for 24 h with concentrations of AMG-47a above 1 µM (Fig. 3E). Finally, to determine whether the observed protection was specific to the necroptotic pathway, we treated U937 and HT29 cells with the apoptotic stimulus of TNF and Smac-mimetic (TS) ± AMG-47a. Although AMG-47a slightly reduced apoptotic death of U937 cells at lower concentrations, this protection was not observed in the dose range required to block necroptosis, and it had no effect on apoptotic death of HT29 cells (Supplementary Fig. 3N, O). Together, these experiments indicate that cells stimulated with less potent necroptotic stimuli and/or those expressing necroptosis effectors at lower abundance are more amenable to AMG-47a protection.

**Table 1:** IC_50_ values of AMG-47a in human cell lines.

Cell line	Necroptotic stimulus	Time (h)	IC_50_ (µM)	95% CI (µM)
U937	TSQ	24	0.29	0.11 to 0.80
		48	0.11	0.07 to 0.18
	TSI	24	1.13	0.67 to 2.03
		48	1.62	0.55 to 5.97
HT29	TSQ	24	0.12	0.03 to 0.32
		48	0.51	0.18 to 1.78
	TSI	24	0.38	0.10 to 1.56
		48	2.44	1.12 to 6.00
THP-1	TSI	24	0.15	0.04 to 0.45
		48	0.32	0.12 to 0.92

IC_50_ values were calculated from a minimum of 3 independent experiments and are derived from the data presented in Fig. 3 and Supplementary Fig. 2A–D.

### AMG-47a inhibits necroptosis downstream of MLKL activation

**Fig. 4:**
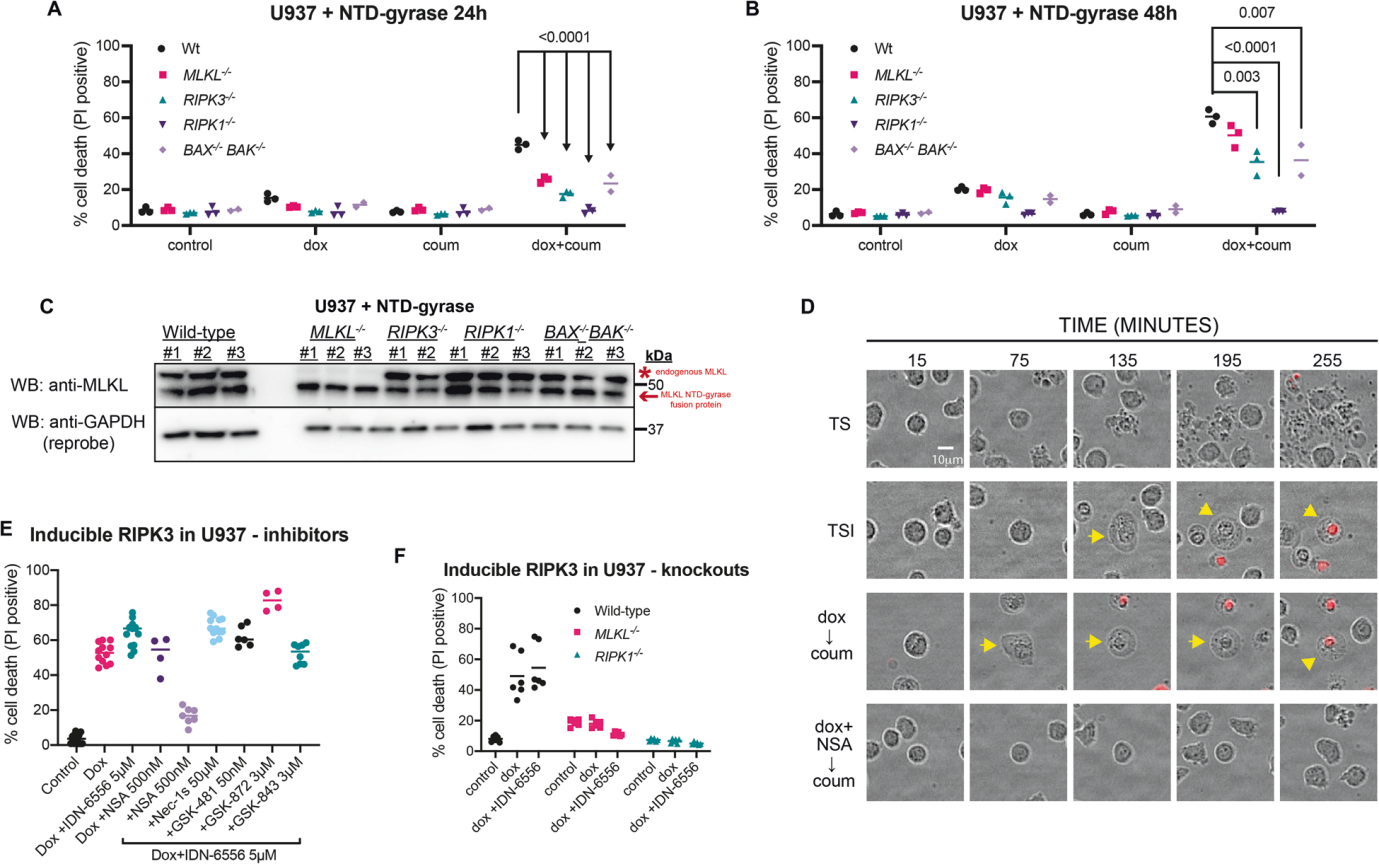
RIPK1 impacts cell death downstream of MLKL activation. **A**, **B** U937 cell lines of various genotypes expressing an inducible MLKL(1-180)-gyrase fusion were treated with doxycycline (to induce expression) and/or coumermycin (to induce dimerisation), or left untreated. After 24 h (**A**) or 48 h (**B**), cells were harvested and assessed for cell death (PI uptake measured using flow cytometry), counting a minimum of 5000 cells. Data represent three independent experiments, with the exception of the *BAX*^*−/−*^*BAK*^*−/−*^ cell lines, where *n* = 2. Statistics were calculated in GraphPad Prism 8, and *p* values are shown where <0.05. **C** The same U937 cells used in panel A and B were treated with doxycycline alone and left overnight. After approximately 16 h, cells were harvested and lysed for analysis of fusion protein expression via western blot. The upper MLKL band (*) represents endogenous MLKL, and the lower band (arrow) represents the MLKL^(1-180)^-gyrase fusion protein. **D** Wild-type U937 were treated with TNF (100 ng/ml), Smac-mimetic (0.5 µM), and caspase inhibitor IDN-6556 (emricasan; 5 µM) (TSI) to induce necroptosis or TNF and Smac-mimetic (TS) alone to induce apoptosis. In parallel, *MLKL*^*−/−*^ U937 expressing an inducible MLKL^(1-180)^-gyrase fusion were treated with doxycycline and coumermycin in the presence or absence of necrosulfonamide (NSA). Images were taken at the specified timepoints using an IncuCyte S3 System to track morphological changes and PI uptake (shown by red). Cells with a necroptotic phenotype (cellular swelling) are indicate with yellow arrows. Three fields were examined per well with representative data shown. **E** Wild-type U937 cells were treated with doxycycline (20 nM) to induce expression of human wild-type RIPK3 in the presence of various cell death inhibitors, as indicated, for 24 h. At the conclusion of the experiment, cells were analysed for PI uptake using flow cytometry, with a minimum of 5000 cells counted. Data represent four or more independent experiments. **F** Wild-type, *MLKL*^*−/−*^ and *RIPK1*^*−/−*^ U937 expressing inducible human wild-type RIPK3 were treated with doxycycline (20 nM) and IDN-6556 (5 µM), doxycycline alone, or left untreated. After 24 h, cell death was measured as PI uptake using flow cytometry. Data are a summary of six independent experiments.

As we had observed that AMG-47a was more effective at blocking necroptosis in human cells, and because human and mouse MLKL have mechanistic differences in the way they kill cells [30, 31, 33, 54, 60, 61], we focused on how AMG-47a inhibited necroptosis at, or downstream of, MLKL activation in human cells. We therefore generated U937 cells that inducibly-expressed regions of the N-terminal killer domain of human MLKL fused to a C-terminal gyrase B domain, which can be forced to dimerise on addition of the bivalent antibiotic coumermycin [62]. In our hands, this is the only system that we have found to induce MLKL-dependent cell death independent of activation of upstream signalling in human cells [30, 31]. We initially tested a human MLKL^(1–180)^ fusion, expressing the full N-terminal domain (NTD) consisting of the four-helix bundle (4HB) and neighbouring two-helix “brace region” of the protein. The dimerised NTD-gyrase construct induced cell death in wild-type U937 cells, as previously reported [30], and also, albeit to a slightly lesser extent, in *MLKL*^−/−^, *RIPK3*^−/−^, and *BAX*^*−/−*^*BAK*^*−/−*^ cells (Fig. 4A–C). IncuCyte imaging of the *MLKL*^*−/−*^ cells expressing this construct confirmed that cells exhibited swelling synonymous with necroptotic cell death during induction which, importantly, could be blocked by treatment with necrosulfonamide (NSA [23]) (Fig. 4D). Surprisingly, this construct was completely unable to kill cells when expressed in *RIPK1*^*−/−*^ U937 cells, even when induced and dimerised over 48 h (Fig. 4B), despite the fusion protein being expressed similarly in all cell lines (Fig. 4C). Furthermore, the brace region of human MLKL contributed to the toxicity of this construct, because a fusion protein without the brace and consisting only of the 4HB domain (MLKL^(1–125)^) could not induce cell death in the absence of endogenous MLKL (Supplementary Fig. 3A).

To test this further, we used another independent system to directly activate MLKL by inducibly expressing human RIPK3 in wild-type U937 cells. To overcome the propensity of RIPK3 to induce apoptosis when overexpressed [63, 64], a caspase inhibitor must be added simultaneously with RIPK3 induction to induce necroptotic cell death. Consistent with these published data, cell death induced by RIPK3 was only blocked by NSA in the presence of a caspase inhibitor (Fig. 4E). Remarkably, inducible RIPK3 necroptosis was also dependent on RIPK1 (Fig. 4F), since *RIPK1*^−/−^ cells were resistant to RIPK3 induced death, although this death could not be blocked by the RIPK1 kinase inhibitors, Nec-1s and GSK-481, or the RIPK3 kinase inhibitors, GSK-843 and GSK-872 (Fig. 4E).

**Fig. 5:**
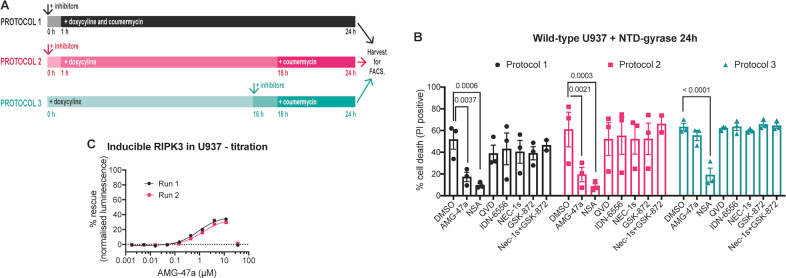
AMG-47a inhibits necroptosis downstream of MLKL activation. **A** Three different experimental protocols were used to assess the ability of AMG-47a to inhibit cell death caused by the expression and dimerisation of the MLKL^(1-180)^-gyrase fusion protein. In protocol 1, inhibitors (or DMSO) were added first, followed by doxycycline and coumermycin together, so that the fusion protein could dimerise on expression. In protocol 2, addition of coumermycin was delayed to allow levels of the fusion protein to accumulate before dimerisation was induced. In protocol 3, the addition of inhibitors (or DMSO) was also delayed, so that it was added after the fusion protein had been expressed for 16 h, but before the addition of coumermycin. In all experimental methods, cells were harvested 24 h after the experiment was initiated. **B** Wild-type U937 cells expressing the MLKL^(1-180)^-gyrase fusion protein were treated using either protocol 1, 2, or 3, as described above (**A**). At the conclusion of the experiment, cells were analysed for PI uptake using flow cytometry, with a minimum of 5000 cells counted. Data represent three independent experiments; bars indicate the mean and error bars indicate standard error of the mean. Statistics were calculated in GraphPad Prism8, and *p* values are shown where <0.05. **C** Wild-type U937 cells with inducible human RIPK3 were treated with increasing doses of AMG-47a in the presence of 40 nM doxycycline, to induce expression, and 5 µM IDN-6556, to block caspase-dependent cell death. After 48 h, cells were harvested and viability assessed using the Cell-Titer Glo 2 (Promega) system. Data represent two independent experiments (four replicates per experiment) and have been normalised against doxycycline plus IDN-6556 (0% viability) and doxycycline plus IDN-6556 plus NSA (100% viability).

Having established MLKL-dependent cell death assays, we used three different protocols to examine whether AMG-47a could block death induced by expression and dimerisation of the NTD-gyrase constructs in U937 (Fig. 5A). In the first protocol, we treated cells with AMG-47a, other known cell death inhibitors, or DMSO, then added doxycycline and coumermycin 1 h later. This protocol promotes dimerisation and activation of the fusion proteins as they are expressed and provides AMG-47a with a long window of action. In the second protocol, we also added AMG-47a prior to induction of expression, but we substantially delayed addition of coumermycin. This allowed levels of the fusion protein to accumulate prior to dimerisation, which, we hypothesised would be a stronger, acute cell death stimulus for AMG-47a to inhibit. Finally, in the third protocol, we expressed the protein overnight in the absence of inhibitors, adding either inhibitor (or DMSO) just prior to addition of coumermycin to determine whether any inhibitory effects were related to the dimerisation step. Importantly, in all protocols cell death was nearly completely blocked by NSA, confirming that in each case the cell death was MLKL-dependent (Fig. 5B, Supplementary Fig. 3B–D). For all experiments, inhibitors were used at doses that were non-toxic for the duration of the experiment (Supplementary Fig. 3E). Based on previous reports that low levels of activated MLKL can be turned over [48, 49, 51, 65, 66], we had anticipated that a slow build-up of active MLKL (protocol 1) might be less toxic to cells than protocol 2. However, we did not observe such a difference (Fig. 5B). Consistent with this, using either protocol 1 or 2, we observed that AMG-47a inhibited cell death in wild-type U937 cell lines expressing the NTD-gyrase fusion protein, although AMG-47a did not block cell death as efficiently as pretreatment with NSA (Fig. 5B). Similarly, pretreatment with AMG-47a reduced cell death when the NTD-gyrase fusion was dimerised in *MLKL*^−/−^, *RIPK3*^−/−^ or *BAX*^−/−^*BAK*^−/−^ U937 cells (Supplementary Fig. 3B–D). However, when we delayed the addition of AMG-47a (protocol 3) we found it was no longer able to protect against cell death in any of the genotypes tested, although NSA was still able to provide near complete protection (Fig. 5B, Supplementary Fig. 3B, C). We did not observe any protection, in any of the genotypes or protocols, when cells were pretreated with the RIPK1 kinase inhibitor Nec-1s or the RIPK3 kinase inhibitor GSK-872, either as a single agent or in combination, with the exception of a small but significant difference when the combination was used in *BAX*^−/−^*BAK*^−/−^ U937 cells (Fig. 5B, Supplementary Fig. 3B–D). We also observed some protection from caspase inhibitors alone in both *MLKL*^−/−^and *RIPK3*^−/−^ U937 cells (Supplementary Fig. 3B, C).

Finally, we assessed the ability of AMG-47a to protect against cell death caused by inducible expression of human RIPK3. In this assay, AMG-47a was protective at doses greater than 400 nM, although, as before, AMG-47a was toxic at 30 μM, the highest dose tested (Fig. 5C). Together, these results support the idea that AMG-47a can inhibit MLKL-induced necroptotic cell death, particularly following treatment with less severe necroptotic stimuli. Furthermore, they suggest that, in human cells, the NTD of MLKL requires RIPK1 to kill, and that AMG-47a can inhibit necroptosis downstream of MLKL activation.

### AMG-47a is a dual RIPK1/RIPK3 inhibitor

**Fig. 6:**
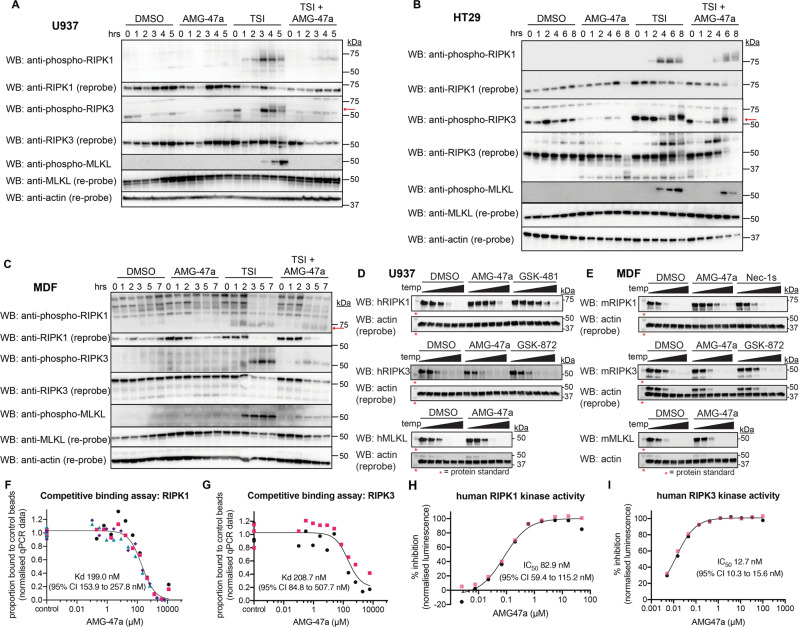
AMG-47a is a dual RIPK1-RIPK3 inhibitor. **A, B, C** U937, HT29 and MDF cell lines were treated with DMSO, AMG-47a (1 µM for U937 and MDF; 2 µM for HT29), TSI, or a combination of AMG-47a and TSI over a time course as indicated in the individual figure panels. At the conclusion of the experiment, cells were harvested and lysed for western blot analysis. All cell lines were assessed for phosphorylation and total protein of the three key necroptosis effector proteins, RIPK1, RIPK3, and MLKL, and total β-actin was used as a loading control. Where multiple bands are present, the specific band of interest is indicated with a red arrow. Data are representative of at least three independent experiments. U937 (**D**) or MDF (**E**) cell lines were treated with AMG-47a at 10 µM, the RIPK1 inhibitors (GSK-481, Nec-1s) or a RIPK3 inhibitor (GSK-872) at 20 µM, or an equivalent amount of DMSO, for 1 h at 37 °C. Cell suspensions were then heated over an increasing temperature gradient (as indicated in the methods) for 3 min. Cells were then lysed, and the amount of remaining soluble protein at each temperature point was analysed using Western blot. Results are representative of two (RIPK1 and RIPK3 in U937) or three (all other analyses) independent experiments. AMG-47a binding to RIPK1 (**F**) and RIPK3 (**G**) was measured in competitive binding assays using the KINOMEscan^®^ Assay Platform (DiscoverX). Graphs were plotted using the raw data supplied from DiscoverX, and a curve was fitted using non-linear regression (GraphPad Prism) to determine the K_d_ and associated error (95% confidence interval). Data represent one (RIPK3) or two (RIPK1) independent experiments, with two runs performed in all experiments and each colour on the graph representing a separate run. All data points were used for the non-linear regression. K_d_ determinations for individual replicates can be found in supplementary data (Supplementary Fig. 4A–F). **H**, **I** AMG-47a was serially diluted from a maximum concentration of 50 µM (RIPK1) or 100 µM (RIPK3), and its ability to inhibit human RIPK1 or RIPK3 kinase activity was assessed using the ADP-Glo Kinase Assay (Promega). The reaction was allowed to proceed for 4 h before readouts were performed, and data were normalised to DMSO (0% inhibition) and either 1 µM GSK-481 (100% inhibition of RIPK1) or 1 µM GSK-872 (100% inhibition of RIPK3). Data are a summary of two independent experiments performed in duplicate, and the IC_50_ and associated error (95% confidence interval) were determined using non-linear regression, as above. Each colour on the graph represents one independent experiment.

Based on the above results, we hypothesized that following a classic necroptotic stimulus AMG-47a might operate both upstream and downstream of MLKL activation. To test this idea, we performed a time-course to examine phosphorylation of RIPK1, RIPK3, and MLKL in the human U937 and HT29 cell lines undergoing necroptosis in the absence or presence of AMG-47a. In both U937 and HT29 cells, we saw a marked reduction of MLKL phosphorylation when cell lines were pretreated with AMG-47a, as well as a delay in both RIPK1 and RIPK3 phosphorylation in U937 cells, however inhibition of RIPK1 and RIPK3 phosphorylation by AMG-47a was less pronounced in HT29 cells (Fig. 6A, B). Additionally, we observed delayed RIPK1 phosphorylation in MDFs pretreated with AMG-47a, as well as a reduction in both RIPK3 and MLKL phosphorylation (Fig. 6C). To determine whether AMG-47a was interacting with these proteins in a cellular context, we performed cellular thermal shift assays (CETSA [67]) in both U937 and MDF cell lines. In both cell lines we found incubation with AMG-47a increased the stability of RIPK1 to a similar or greater extent than known RIPK1 inhibitors GSK-481 and Nec-1s, suggesting that AMG-47a binds to RIPK1 in cells (Fig. 6D, E). However, neither RIPK3 nor MLKL stability was affected by the presence of AMG-47a in either cell line, suggesting that they are not direct targets of AMG-47a in cells.

To better understand the nature of these interactions, we determined the K_d_ of the interaction between AMG-47a and the necroptotic effector proteins using the DiscoverX competitive kinase binding assay [68]. We found that AMG-47a bound to both RIPK1 (K_d_ 199 nM; 95% CI 154–258 nM; Fig. 6F, Supplementary Fig. 4A–D) and RIPK3 (K_d_ 209 nM; 95% CI 85–508 nM; Fig. 6G, Supplementary Fig. 4E, F), confirming that AMG-47a could influence their ability to bind ATP. As described previously [57], we also found AMG-47a bound VEGFR2, with 50% binding observed at 50 nM and over 90% binding at 500 nM. In contrast, no binding to human MLKL was detected at 12 μM, the maximum dose tested. Finally, we assessed whether AMG-47a binding affected RIPK1 and RIPK3 kinase activity. We found that AMG-47a was able to inhibit both RIPK1 and RIPK3 kinase activity in enzymatic assays, with IC_50_ values of 83 nM (95% CI 59–115 nM) and 13 nM (95% CI 10–16 nM), respectively (Fig. 6H, I). Together, these results indicate that AMG-47a inhibits MLKL induced necroptosis by targeting a RIPK1/MLKL function in cells and inhibits the kinase activity of both RIPK1 and RIPK3.

## Discussion

In this study, we identified AMG-47a as an inhibitor of necroptosis. While it functioned to block necroptosis induced by activated forms of MLKL, such as the Q343A mouse MLKL mutant and dimerised human MLKL NTD, we were unable to find evidence that it interacts directly with MLKL. Instead, our data indicate that AMG-47a interacts with both RIPK1 and RIPK3 to inhibit necroptosis. Together with our genetic data, this strongly suggests that constitutively-activated forms of human MLKL rely on RIPK1 and, to a lesser extent, RIPK3 for their full cytotoxic activity. Notably, while AMG-47a competed with ATP binding to both RIPK1 and RIPK3 and inhibited their kinase activity in vitro, other documented inhibitors of these kinases did not prevent activated MLKL killing. Therefore, the role of RIPK1 and RIPK3 in activated MLKL killing appears to be independent of their kinase activity. These data substantially revise our understanding of necroptosis as a linear pathway and expose the limitations of classic genetic approaches as a means to understand such pathways. Thus, if RIPK1 is required both upstream and downstream of MLKL activation, only the epistatic upstream effects would be revealed by genetic deletion of these effectors. Such an interpretation of our results fits with recent findings that suggest a combination of regulators determines whether cells undergo necroptosis or apoptosis rather than a simple linear pathway [20–22].

AMG-47a was identified as an Lck inhibitor, which inhibited IL-2 production in human and mouse cells [57]. However, binding of AMG-47a to Lck is unlikely to contribute towards its protective effects against necroptosis found here, as Lck expression is known to be very low in HT29 cells [69, 70]. In addition to binding of Lck, AMG-47a was also found to bind kinases involved in cell death pathways, including MAPKs, such as p38α, at low nanomolar concentrations and JNK1-3 at mid nanomolar concentrations, with AMG-47a shown to reduce ERK1/2 phosphorylation in A375 human melanoma cells [71]. However, we only observed a limited capacity of AMG-47a to block extrinsic apoptosis. In the subset of lines that we tested, AMG-47a was a more effective inhibitor of necroptosis in human (2 leukemic and one colorectal) than mouse (3 leukemic and 2 fibroblast) cell lines, which may be related to the higher toxicity of AMG-47a to mouse cells. However, despite the differing levels of protection, AMG-47a was able to reduce MLKL phosphorylation and stabilise RIPK1 equally in both mouse and human cell lines. Additionally, AMG-47a protected against necroptosis in murine AML cell lines with low levels of necroptosis effectors or in MDF cells treated with a less severe necroptotic stimulus. Together, this suggests the differences in response to AMG-47a between mouse and human cells may be more related to subtle differences in the regulation of necroptosis between these species.

Current models for how MLKL kills downstream of RIPK3 phosphorylation posit that phosphorylation causes a conformational change in MLKL that exposes its NTD [26, 35, 36, 65]. Expression of the mouse MLKL NTD is sufficient to kill mouse cells, suggesting that it contains the necessary oligomerisation and membrane permeabilisation activities required to kill cells that are normally held in check by the pseudokinase domain [25–27, 72]. This simple mechanism does not translate to human cells because phosphomimetic mutants of MLKL, or expression of the NTD alone, are inadequate to kill human cells [30, 31]. However, stimulus independent cell death can be induced in human cells when the N-terminal regions of human MLKL are fused to a dimerisation domain [30], supporting the idea that there are additional regulatory processes between phosphorylation of MLKL and cell death in the human necroptotic pathway that are not present in mouse cells. Unexpectedly, we found that death mediated by forced dimerisation of the human MLKL NTD was completely dependent on the presence of the upstream necroptotic effector protein, RIPK1, and to a lesser extent on RIPK3. These findings, combined with recent data showing RIPK1 co-localises with MLKL after phosphorylation in human HT29 cells [32], suggest that a prolonged association with necrosome components is important for progression of necroptosis after MLKL is phosphorylated by RIPK3 in human cells. The extent to which human cell lines more broadly rely on RIPK1 downstream of MLKL NTD forced dimerization for cell death remains the subject of ongoing interest. Further interrogation of the role of RIPK1 downstream of MLKL activation awaits the generation of necroptosis-competent cell lines, such as THP1 [73] or Molt4 [32], in which *RIPK1*, *RIPK3* or *MLKL* have been knocked out.

Interestingly, our finding that RIPK1 was a target of AMG-47a suggests that it may block NTD-gyrase-mediated cell death through either direct or indirect interaction with RIPK1. Because the known RIPK1 kinase inhibitor, Nec-1s, was unable to block NTD-gyrase-mediated cell death, this suggests that RIPK1 likely plays a scaffolding role, such as nucleating assembly of necrosomes in the cytoplasm, as platforms for activation of the downstream effectors, RIPK3 and MLKL. Further validation of the kinase-independent role of RIPK1 downstream of MLKL activation awaits future generation of necroptosis-competent cell lines expressing kinase-dead mutant RIPK1. Although RIPK1 has an established role as an important apical component in necroptotic signalling, studies in mouse cells highlight a more complex involvement of RIPK1, including restricting necroptosis in particular contexts, especially during development [74–77]. Additionally, there have also been a number of reports indicating that necroptosis can proceed in the absence of RIPK1 in mouse cells [78–80], particularly in the case of interferon signalling, where the RHIM-containing protein ZBP-1/DAI was proposed to interact with RIPK3 to induce necroptosis [81, 82]; whether this holds true in human cell necroptosis has not been investigated. Our findings indicate the involvement of RIPK1 in the progression of necroptosis may not be analogous between species and indicates an obligate role downstream of MLKL activation in human cells. This is supported by our finding that death caused by human RIPK3 overexpression was also reliant on the presence of RIPK1. This contrasts previous findings in mouse cell lines, where RIPK3 overexpression could induce TNF-dependent necroptosis in the absence of RIPK1 [78]. Interestingly, like the NTD-gyrase experiments, killing induced by human RIPK3 overexpression could not be blocked by established RIPK1 kinase inhibitors, but could be inhibited by AMG-47a, suggesting a similar mechanism for RIPK1 may be involved in both contexts. Notably, both Nec-1s and GSK-481 are classified as Type III inhibitors and bind the same allosteric region of RIPK1 overlapping the ATP binding site [83], whereas AMG-47a is a Type II inhibitor and bind within the ATP binding site of Lck [57]. It is possible that these differences in binding mode could lead to structural differences in RIPK1 which may change how RIPK1 interacts with MLKL in the presence of different inhibitors.

**Fig. 7:**
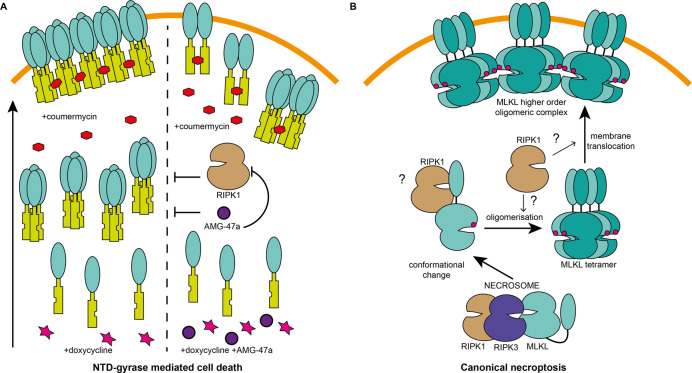
Model for a role of RIPK1 in necroptosis downstream of MLKL activation. **A** The cell death induced by forced dimerisation of the human MLKL NTD involves two distinct processes. In the first, MLKL can self-associate into discrete oligomers on addition of doxycycline. In the second, addition of coumermycin promotes the assembly of large multimeric complexes. AMG-47a is unable to inhibit the second process, thus we propose it prevents membrane attachment, translocation, or oligomerisation of the human MLKL NTD through its activity as a kinase inhibitor. As AMG-47a binds RIPK1, and as RIPK1 is required for this form of cell death, we propose the primary target of AMG-47a is RIPK1 in this context. **B** When a cell receives a necroptotic stimulus, a number of key events occur, beginning with assembly of a necrosome containing RIPK1, RIPK3, and MLKL. This triggers a number of events, beginning with the phosphorylation of MLKL by RIPK3, which enables a conformational change in MLKL that allows oligomerisation, translocation to cellular membranes, and ultimately cell death. Our data suggest an additional role for RIPK1 in human necroptosis by supporting the conformational change, oligomerisation, or membrane translocation of MLKL following phosphorylation by RIPK3.

The data reported herein have enabled us to propose a revised model of human necroptotic signalling downstream of MLKL activation (Fig. 7). Our NTD-gyrase experiments suggest two distinct processes: assembly of the MLKL NTD into discrete oligomers, likely through interactions mediated by the brace region of the NTD; and addition of coumermycin acts to link the MLKL oligomers into large multimeric complexes that can destabilise cell membranes and induce cell death (Fig. 7A). Our data suggest that AMG-47a prevents cell death mediated by forced dimerisation of the human MLKL NTD by inhibiting the formation of the MLKL NTD into discrete oligomers, and/or by inhibiting the translocation of these oligomers to cell membranes. This mechanism of action contrasts the MLKL NTD inhibitor, NSA, which was able to block cell death when introduced both before expression and before dimerisation. These results, together with the reliance of human MLKL NTD-gyrase and human RIPK3 overexpression on RIPK1 for necroptotic signalling, indicate that, in the context of the human necroptotic pathway, RIPK1 may have an additional role in activating the MLKL NTD when disengaged from the pseudokinase domain post-phosphorylation (Fig. 7B). Whether this supports the conformational change of MLKL post-phosphorylation, mediates MLKL oligomerisation, or facilitates translocation of MLKL to cellular membranes is yet to be determined. A dual role of RIPK1 at the apex of the human necroptosis pathway and downstream of RIPK3/MLKL activation, as identified here, helps provide further insight into the mechanistic differences in necroptosis signalling that occur downstream of MLKL phosphorylation in mouse and human cells.

## Materials and methods

### Antibodies, chemicals, and reagents

The following primary antibodies were used in this study: rat anti-MLKL clone 3H1 (available as MABC604 from Merck Millipore; 1:1000) was produced in-house as previously described [25]; rabbit anti-human MLKL pS358 (ab187091, Abcam, Cambridge, United Kingdom; 1:4000); rabbit anti-mouse MLKL pS345 (ab196436, Abcam; 1:1000); rat anti-human RIPK3 (clone 1H2; available as MABC1640 from Merck Millipore; 1:1000) and rat anti-mouse RIPK3 (clone 8G7; available as MABC1595 from Merck Millipore; 1:1000) produced in-house as previously described [3, 84]; rabbit anti-RIPK3 (2283, ProSci; 1:1000); rabbit anti-human RIPK3 pS227 (ab209384, Abcam; 1:2000); rabbit anti-mouse RIPK3 pT231/pS232 (GEN135-35-9, kindly supplied by Genentech, South San Francisco, CA, USA [74]; 1:2000); mouse anti-RIPK1 (610458, BD Transduction Laboratories, San Jose, CA, USA; 1:1000); rabbit anti-RIPK1 (3493, Cell Signalling Technology, Danvers, MA, USA; 1:1000); rabbit anti-human RIPK1 pS166 (65746, Cell Signalling Technology; 1:1000); rabbit anti-mouse RIPK1 pS166 (31122, Cell Signalling Technology; 1:1000); rabbit anti-mouse CASP8 (4927, Cell Signalling Technology; 1:1000); rabbit anti-BAX (2772, Cell Signalling Technology; 1:1000); rabbit anti-BAX (06-536, Upstate Biotechnology, Lake Placid, NY, USA; 1:1000); rabbit anti-GAPDH (2118, Cell Signalling Technology; 1:1000); and mouse anti-β-Actin (A1978, Sigma-Aldrich; 1:3000). Polyclonal goat anti-mouse (1010-50), anti-rabbit (4010-05), and anti-rat (3010-50) HRP-conjugated secondary antibodies were all used at 1:5000 (SouthernBiotech, Birmingham, AL, USA). For Cellular Thermal Shift Assay (CETSA) experiments, chicken anti-β-actin-HRP (Santa Cruz Biotechnology, cat #sc-47778 HRP) was also used at 1:5000.

The following chemicals and biological reagents were used in this study: pan-caspase inhibitor QVD-OPh (HY-12305, MedChemExpress, Monmouth Junction, NJ, USA); Necrostatin-1s (Nec-1s; Calbiochem 504297, Merck Millipore, Darmstadt, Germany); GSK-481 (made by SYNthesis MedChem, Parkville, Australia); GSK-843 (SYN-5482, SYNkinase, Parkville, Australia); GSK-872 (SYN-5481, SYNkinase); and necrosulfonamide (NSA; 480073, Merck Millipore). AMG-47a for the phenotypic screen was produced by Compounds Australia, and for all other experiments, AMG-47a used was purchased through SYNkinase (SYN-1007) or MedChemExpress (HY-18303); all behaved analogously in side-by-side assays. The Smac-mimetic, Compound A [85], and pan-caspase inhibitor IDN-6556/emricasan were supplied by TetraLogic Pharmaceuticals. Human recombinant IL-3 was purchased from R&D Systems (203-IL, Minneapolis, MN, USA), and human recombinant TNF (hTNF-Fc) was produced in-house as previously described [86].

### Phenotypic screen

A library of 276 kinase inhibitors with known targets was used for a phenotypic screen as previously described [39]. In brief, wild-type or *Mlkl*^*−/−*^ murine dermal fibroblasts (MDFs) expressing inducible MLKL^Q343A^ were seeded into 384-well plates pre-prepared with the compounds at 1 µM by Compounds Australia (Nathan, Australia) at 2000 cells/well. After 2 h pre-incubation with compounds, cell death was induced by addition of TNF (100 ng/mL), Smac-mimetic (0.5 µM) and QVD-OPh (5 µM) (TSQ) in the case of wild-type MDFs, and by addition of 1 µg/ml doxycycline to induce expression of MLKL^Q343A^ in *Mlkl*^*−/−*^ MDFs. Cell viability was determined using the CellTiter-Glo^®^ Luminescent Cell Viability Assay (Promega, Madison, WI, USA) after 24 h of treatment and normalised to DMSO-treated controls (100% viability) and TSQ/doxycycline only controls (0% viability).

### Cell culture and AMG-47a titrations

Human histiocytic lymphoma U937 and human acute monocytic leukaemia THP-1 cell lines (ATCC) were cultured in RPMI media (produced in-house) supplemented with 8–10% foetal calf serum (FCS; Sigma-Aldrich). Murine acute myeloid leukaemia (AML) cell lines were cultured in Iscove’s modified Dulbecco medium (IMDM; in-house) with 10% FCS and additional IL-3 (3 ng/ml). MLL-AF9, MLL-AF9-Ras, MLL-ENL and HoxA9-Meis1 AMLs were generated as previously described using retroviral constructs [58, 59]. For cell death assays, cells were seeded into 96-well plates at 5 × 10^4^ cells/well and treated with increasing concentrations of AMG-47a (as stated in legends), or 0.5% v/v DMSO (Sigma-Aldrich) in control wells. After 2 h, cell death was induced in U937 and THP-1 using TNF (100 ng/ml), Smac-mimetic (0.5 µM) and a caspase inhibitor (QVD-OPh at 10 µM or IDN-6556 at 5 µM) (TSQ/TSI); for murine AML lines, Smac mimetic and IDN-6556 (SI) were used alone to induce cell death, at the same concentrations as above. After a further 24 h, cell death was assessed by measuring % propidium iodide (PI; 1 µg/ml) uptake using flow cytometry, counting a minimum of 5000 cells.

Human colorectal adenocarcinoma HT29 (ATCC), murine RAW 264.7 macrophage (ATCC), mouse embryonic fibroblast (MEF) and MDF cell lines were cultured in Dulbecco’s modified Eagle’s medium (DMEM; in-house) with additional FCS (8–10%). MDFs were generated from the dermis of wild-type, *Mlkl*^*−/−*^, *Ripk3*^*−/−*^, and *Casp8*^*−/−*^*Mlkl*^*−/−*^ mice and immortalised as previously described [25, 87–89]. *Ripk1*^*−/−*^*Mlkl*^*−/−*^ MDF cell lines generated for this study were harvested from the dermis of *Ripk1*^*−/−*^*Mlkl*^*−/−*^ P1 mice, which have been described previously [75], and were immortalised using SV40 large T antigen-expressing lentivirus using analogous methods. Wild-type and *Bax*^*−/−*^*Bak*^*−/−*^ MEFs were a gift from Grant Dewson and produced as previously described [90, 91]. *Ripk1*^*−/−*^ MEFs were generated from E13.5 embryos [92]. For cell death assays, MDFs/MEFs and HT29 cells were seeded into 48-well plates at 2.5 × 10^4^ or 5 × 10^4^ cells/well respectively, and RAW cells were seeded into 24-well plates at 5 × 10^4^ cells/well. After one (MDF, MEF and RAW cells) or two (HT-29) nights for settling, cells were treated with AMG-47a or DMSO, then stimulated for cell death after 2 h with TSQ or TSI, and assessed for PI uptake using flow cytometry after a further 24 h, as described for above cell lines.

### Cell death assays using constitutively-activated MLKL variants

U937 cells stably transduced with MLKL expression constructs were cultured in RPMI media as described above, with the addition of 2 µg/ml puromycin to maintain selection of cells containing the transgene. For these experiments, we used previously-described human MLKL^(1-156)^- or MLKL^(1-180)^-gyrase fusion constructs [30], although the cell lines used in this study were newly generated. Expression was induced using doxycycline (20 ng/mL), and dimerisation was induced using the divalent antibiotic coumermycin (700 nM). Inhibitors, where used, were at the following concentrations: AMG-47a, 1 µM; QVD-OPh, 10 µM; IDN-6556, 5 µM; Nec-1s, 5 µM; GSK-872, 20 µM; NSA, 1 µM. All experiments were conducted in 96-well plates seeded at a cell density of 5 × 10^4^ cells/well, and cell death was assessed at the end of the experiment (24 h or 48 h) as %PI uptake using flow cytometry, as described above.

### Western blot analyses

For each experiment, 1 × 10^6^ cells were lysed in 100 µL of 2× Laemmli buffer (4% w/v SDS, 20% v/v glycerol, 120 mM Tris-Cl pH 6.8), heated to 95 °C for 10 min, then 15–25 µL of lysate was loaded into 4–15% TGX gels (Bio-Rad Laboratories, Hercules, CA, USA) and separated via SDS-PAGE. After separation, samples were transferred to PVDF membranes (Merck Millipore), with the exception of samples that required probing for murine phosphorylated MLKL, which were transferred to nitrocellulose membranes (Cytiva, Marlborough, MA, USA). After transfer, membranes were blocked with 5% w/v skim milk powder in PBS-T (0.1% v/v Tween-20), then incubated overnight at +4 °C in the relevant primary antibody in PBS-T with 2% w/v BSA and 0.02% w/v sodium azide. The following day, membranes were washed, then incubated in the relevant secondary antibody diluted in 5% skim milk in PBS-T for approximately 1 h at room temperature. Membranes were washed again, then visualised on a ChemiDoc^TM^ Gel Imaging System (Bio-Rad Laboratories) using Immobilon Western Chemiluminescent HRP Substrate (Merck Millipore). Concentrations of various antibodies are listed above. All full, uncropped Western blots from this study are included as Supplemental Material.

### Cellular Thermal Shift Assay (CETSA)

MDF and U937 cells were seeded to achieve 1.5 × 10^6^ cells per temperature point. After treatment with AMG-47a at 10 µM, GSK-481, GSK-872 or Nec-1s at 20 µM, or an equivalent amount of DMSO, cells were incubated for 1 h at 37 °C, then pelleted, washed, and resuspended in 650 μL DPBS (Gibco, Thermo Fisher Scientific, Waltham, MA, USA) containing cOmplete^TM^ EDTA-free Protease Inhibitor Cocktail (Roche, Basel, Switzerland). For each temperature point as indicated below, 100 µL of these live cell suspensions were heated for 3 min in a T100 Thermal Cycler (Bio-Rad), cooled at room temperature for 3 min, snap frozen in liquid N_2_ and stored at −80 °C overnight. After a single freeze-thaw, soluble and precipitated protein were separated by centrifugation at 17,000 x *g* for 30 min at 4 °C, and the soluble protein fractions were resolved by SDS-PAGE and analysed by Western Blot, as described above, with the exception that proteins were transferred to a nitrocellulose membrane using the iBlot Dry Blotting System (Invitrogen, Thermo Fisher Scientific).

Temperature gradients were optimised for each cell line and each protein of interest. For U937, the temperature gradients (°C) used for each human protein were: RIPK1 (44.0, 44.7, 45.9, 47.9, 50.2, 51.9), RIPK3 (44.7, 45.9, 47.9, 50.2, 51.9, 53.2), and MLKL (46.6, 47.7, 49.5, 51.6, 53.2, 54.3). For MDF, the temperature gradients (°C) used for each mouse protein were RIPK1 (43.9, 45.9, 48.2, 49.9, 51.2, 52.0), RIPK3 (48.9, 50.9, 53.2, 55.0, 56.2, 57.0), and MLKL (45.3, 47.2, 49.4, 51.0, 52.2, 53.0).

### In vitro kinase binding assay

Binding of AMG-47a to RIPK1, RIPK3 and MLKL was assessed using the KINOMEscan^®^ Assay Platform (Eurofins DiscoverX, Fremont, CA, USA [68]). In brief, AMG-47a binding to each target was assessed using a competitive binding assay with a compound dilution series with a maximum concentration of 12 µM. Binding of AMG-47a to VEGFR-2, previously described as an interactor [57], was also assessed as a positive control at two discrete concentrations: 50 nM and 500 nM.

### RIPK3 and RIPK1 kinase activity assays

Recombinant wild-type human RIPK3^(2-311)^ and RIPK1^(2-372)^ were expressed in *Sf*21 insect cells as TEV protease-cleavable N-terminal His_6_ fusions and purified as previously described [3]. The kinase activity assay was conducted in 384-well plates using the ADP-Glo^TM^ Kinase Assay (Promega). In brief, a 10-point 3-fold dilution series of AMG-47a was prepared (maximum concentration 50 or 100 µM for RIPK1 and RIPK3, respectively) and combined with either 10 nM human RIPK3^(2-311)^ or 200 nM human RIPK1^(2-372)^ and 10 µM ATP in a protein-specific buffer (RIPK3 buffer: 50 mM NaCl, 50 mM HEPES, pH 7.4, 30 mM MgCl_2_, 0.05% w/v BSA, 0.01% v/v Tween-20, 1 mM DTT; RIPK1 buffer: 50 mM NaCl, 50 mM TRIS, pH 7.4, 30 mM MgCl_2_, 0.05% w/v BSA, 0.01% v/v Triton-X, 1 mM DTT). After a 4 h incubation at room temperature the reaction was stopped with ADP-Glo reagent, then incubated for a further 40–60 min. Kinase Detection Reagent was then added, and the reaction was incubated at room temperature for a further 30–60 min. Luminescence was measured using an Envision plate reader (Perkin Elmer), and raw luminescence data were normalised to DMSO (0% inhibition) and either 1 µM GSK-872 (for RIPK3; 100% inhibition) or 1 µM GSK-481 (for RIPK1; 100% inhibition).

### Inducible RIPK3 cell death assays

A cDNA encoding wild-type human RIPK3 was cloned into the doxycycline-inducible pF TRE 3 G PGK puro expression vector as previously described [25], and introduced into wild-type, *MLKL*^*−/−*^ and *RIPK1*^*−/−*^ U937 cells using lentiviral transduction as previously described [30]. Optimisation experiments were performed in 24- and 96-well plates, with U937 cells seeded at 1 × 10^5^ and 2 × 10^4^ cells/well respectively in the presence of 20 nM doxycycline to induce expression. Cells were treated with IDN-6556, NSA, Nec-1s, GSK-481, GSK-872, GSK-843, or an equivalent amount of DMSO, as indicated in figure legends. After 24 h, cells were harvested, and death analysed by PI uptake using flow cytometry.

Analysis of the ability of AMG-47a to block cell death caused by induction of human RIPK3 was performed in 384-well plates, seeding at 2000 cells/well. A 10-point, 3-fold dilution series of AMG-47a was prepared (maximum end concentration 36 µM), and cells were preincubated with AMG-47a or DMSO, with 5 µM of IDN-6556. After 2 h, expression of the transgene was induced by addition of 40 nM doxycyline to induce cell death. Cells were incubated for 48 h at 37 °C, after which cell viability was assessed using the CellTiter-Glo^®^ 2.0 Cell Viability Assay (Promega) as per the manufacturer’s instructions, with luminescence data collected using an Envision plate reader (Perkin Elmer, Waltham, MA, USA).

### IncuCyte cell death assay

U937 cells were seeded at 3 × 10^4^ cells/well into 48-well plates in RPMI with 8% FCS and left to settle overnight; *MLKL*^*−/−*^ cells with inducible MLKL^(1-180)^-gyrase were incubated overnight with 20 ng/ml doxycycline, either in the presence or absence of 1 µM necrosulfonamide (NSA). Just prior to transfer to the IncuCyte instrument, cells expressing MLKL^(1-180)^-gyrase were treated with 700 nM coumermycin to induce dimerisation and, in conjunction, wild-type U937 were treated with TNF (100 ng/mL), the Smac-mimetic Compound A (500 nM) and the pan-caspase inhibitor IDN-6556 (5 μM) to induce necroptosis, TNF and Smac-mimetic alone to induce necroptosis, or left untreated. Finally, all cells were treated with 1 µg/ml propidium iodide and moved into the Incu-Cyte S3 System (Essen Bioscience) and imaged initially after 15 min (to allow the cells to settle), then every 30 min thereafter, on a ×20 objective using default bright-field and red channel settings, with three distinct fields obtained per well. At the conclusion of the experiment, micrographs were examined using ImageJ v.1.53n [93] in a blinded manner for morphological signs of either necroptosis or apoptosis over time.

### Data collection and analysis

Flow cytometry data were collected using a BD FACSCalibur^TM^ analyser (BD Biosciences, Franklin Lakes, NJ, USA) and analysed using FlowJo^TM^ for Mac v10.6 (Becton Dickinson and Company, Franklin Lakes, NJ, USA). For all experiments, data was plotted using GraphPad Prism8 (GraphPad Software, San Diego, CA, USA) and has been displayed as individual data points with the mean.

All statistics were generated in GraphPad Prism8. Where *K*_*d*_ or IC_50_ were determined (cell death assays, kinase binding assays, kinase activity assays), the best fit curve was generated using an inhibitor vs response (three parameters) model of linear regression. *K*_*d*_/IC_50_ values reported were generated from these analyses. For analysis of MDF cell death assays, we used a standard paired *t*-test. For NTD-gyrase experiments comparing different genotypes we used one-way ANOVA, followed by Dunnet’s multiple comparison test. For the NTD-gyrase experiments comparing the effects of different drugs in the same genotype, we performed repeated measures (randomised block) ANOVA, followed by Dunnet’s multiple comparison test. Where there were missing data, a mixed model method comparable to repeated measures ANOVA was used instead, which, in the absence of missing values, gives the same *P* values and multiple comparisons tests as repeated measures ANOVA, and can be interpreted comparably to this analysis where data are incomplete.

## Supplementary information

Supplementary Figures 1-4

Uncropped western blots

Author checklist file

## Data Availability

All primary data are available from the corresponding authors upon request.
