# Supplementary material for: The Lck inhibitor, AMG-47a, blocks necroptosis and implicates RIPK1 in signalling downstream of MLKL
Source: Cell Death Dis. 2022 Apr 1;13(4):291. doi: 10.1038/s41419-022-04740-w (PMC8976052; doi:10.1038/s41419-022-04740-w)
Supplement: Supplementary file 1 — Supplementary Figures 1-4 [file 41419_2022_4740_MOESM1_ESM.pdf]

SUPPLEMENTARY FIGURES AND FIGURE LEGENDS

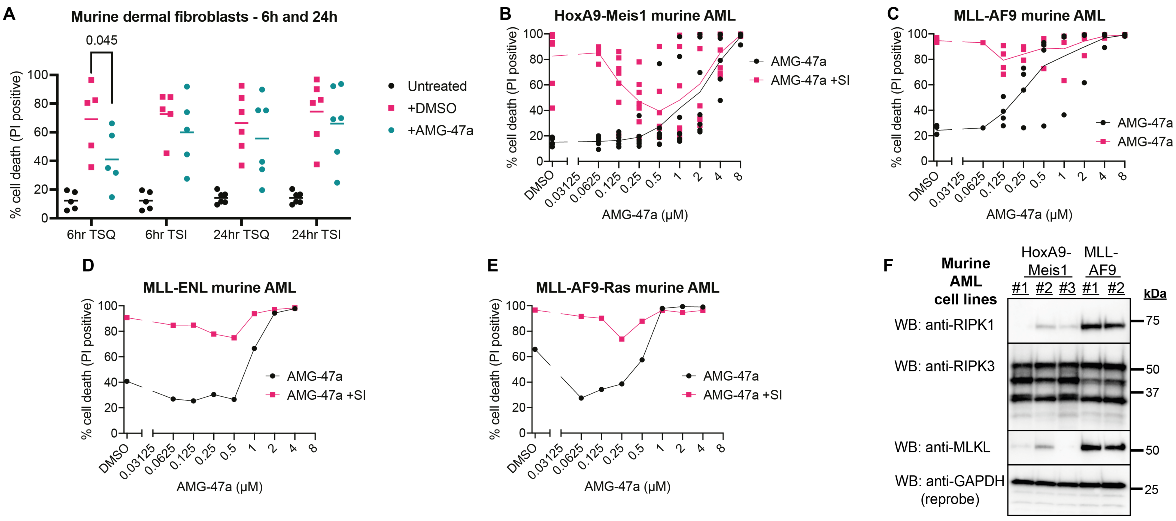

Supplementary Figure 1. – (relates to Figure 1. AMG-47a is a novel necroptosis inhibitor identified in mouse cells.) (A) This panel is the raw data corresponding to normalised data presented in **Fig. 1B**. Wild-type MDFs were treated with AMG-47a (1µM) or an equivalent amount of DMSO, then challenged with TSQ or TSI (TNF (100 ng/ml), Smac mimetic (0.5 µM), and caspase inhibitor IDN-6556 (emricasan; 5 µM)) to induce necroptotic cell death or left untreated. After 6h or 24h PI uptake was measured using flow cytometry as above. Statistics were calculated in GraphPad Prism8, and p values are shown where <0.05. **(B-E)** AMG-47a was assessed in murine AML cell lines in 2-fold dilution series for both toxicity (AMG-47a alone), and its ability to protect against induction of necroptosis **(B-E)** using SI (Smac mimetic (0.5 µM) and IDN-6556 (emricasan; 5 µM)). Cell death was assessed 24 h after treatment by measuring propidium iodide (PI; 1 µg/ml) uptake using flow cytometry, with a minimum of 5000 cells counted. Data represent one **(D, E)**, four **(C)**, or seven **(B)** independent biological replicates, and lines represent the mean (**B, C**) or individual replicates (**D, E**). **(F)** Equivalent amounts of different murine AML cell lines were lysed and assessed via western blot for the levels of the three key effector proteins of necroptosis under baseline (untreated) conditions. Cell lines analysed represent those used in death assays (**B, C, Fig. 1D, E**), and represent two (MLL-AF9) or three (HoxA9-Meis1) individual cell lines. Western blots were performed twice using lysates collected at two different times, and data are representative of these results.

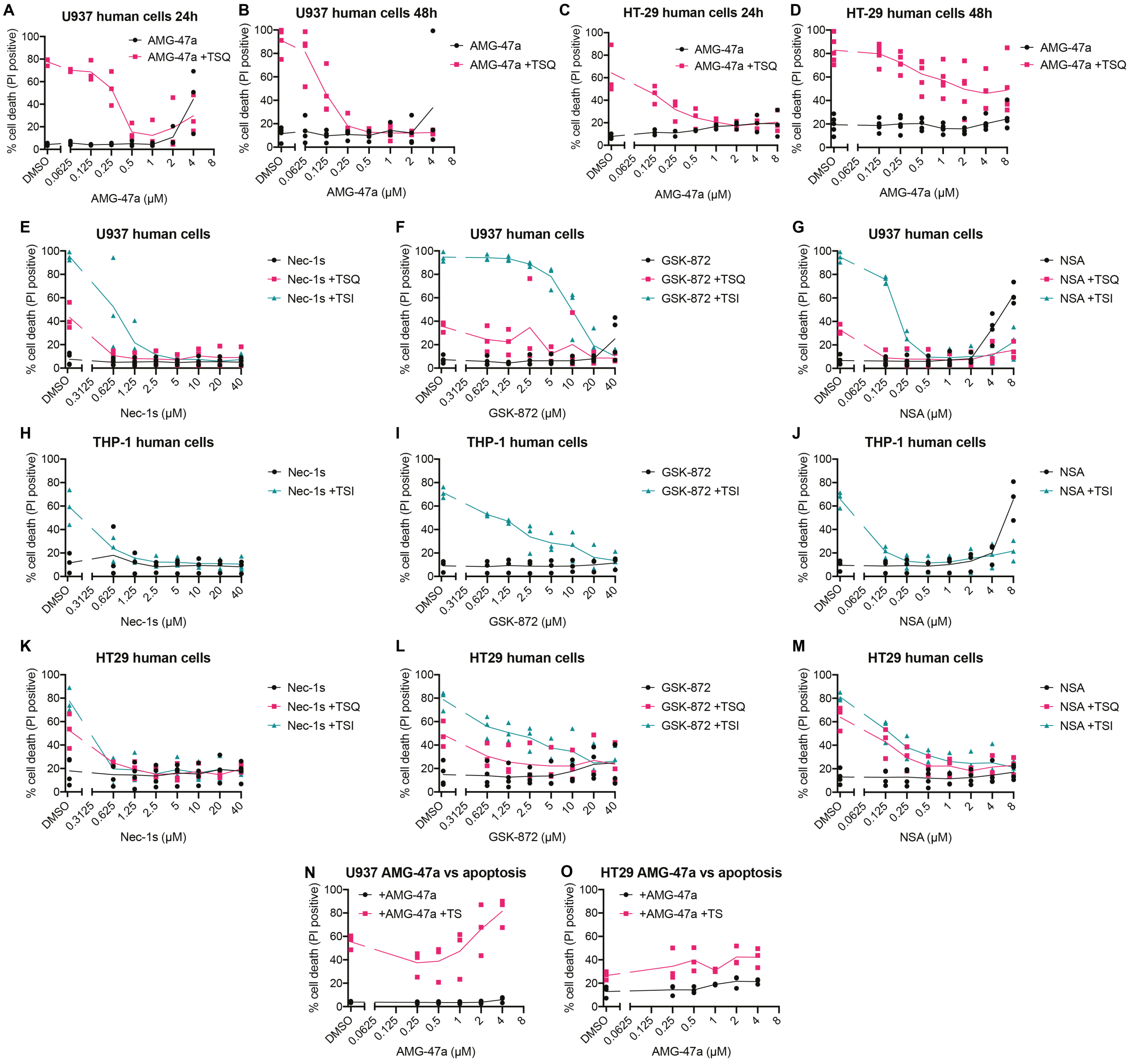

Supplementary Figure 2. – (relates to Figure 3. AMG-47a is a strong necroptosis inhibitor in human cell lines.) (A-D) AMG-47a was assessed in human U937 and HT29 cell lines in 2-fold dilution series for both toxicity (AMG-47a alone), and its ability to protect against induction of necroptosis **(A-D)** using TNF (100 ng/ml), Smac mimetic (0.5 µM), and the caspase inhibitor QVD-OPh (10 µM) (TSQ) for 24 or 48 h. Cell death was assessed by measuring propidium iodide (PI; 1 µg/ml) uptake using flow cytometry, with a minimum of 5000 cells counted. **(E-M)** Necroptosis inhibitors targeting RIPK1 (Necrostatin-1s; Nec-1s), RIPK3 (GSK-872), and MLKL (necrosulfonamide; NSA) were assessed in U937, THP-1 and HT29 human cell lines in 2-fold dilution series for both toxicity (inhibitor alone), and their ability to protect against induction of necroptosis using TNF (100 ng/ml), Smac mimetic (0.5 µM), and either the caspase inhibitor IDN-6556 (5 µM) (TSI) or QVD-OPh (10 µM) (TSQ). Cell death was assessed 24 h after treatment using flow cytometry, as above. **(N, O)** AMG-47a was assessed, as above, for its ability to protect against

apoptosis using TNF and Smac mimetic alone (TS) in U937 and HT29 cells at 24 h. Data represent three (**A-C, E-O**) or four (**D**) independent biological replicates, and lines represent the mean.

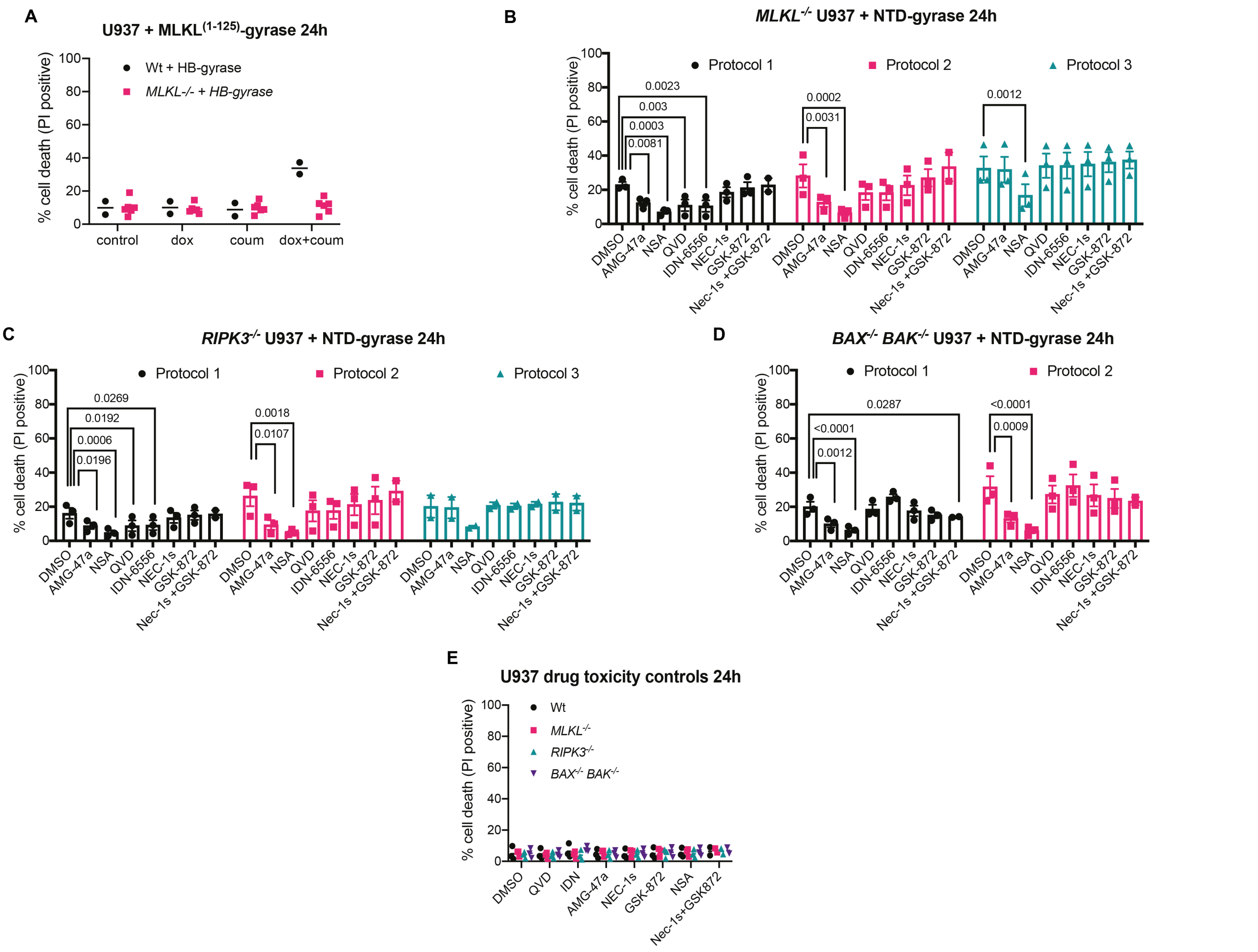

Supplementary Figure 3. – (relates to Figure 4. RIPK1 impacts cell death downstream of MLKL activation and Figure 5. AMG-47a inhibits necroptosis downstream of MLKL activation.) (A) Wild-type and *MLKL*$^{-/-}$ U937 cell lines expressing an inducible MLKL$^{(1-125)}$-gyrase fusion were treated with doxycycline (to induce expression) and/or coumermycin (to induce dimerisation), or left untreated. After 24 h, cells were harvested and assessed for PI uptake using flow cytometry, counting a minimum of 5000 cells. Data represent two (wild-type) or six (*MLKL*$^{-/-}$) independent experiments. **(B, C, D)** *MLKL*$^{-/-}$ **(B)**, *RIPK3*$^{-/-}$ **(C)**, or *BAX*$^{-/-}$*BAK*$^{-/-}$ **(D)** U937 cells expressing the MLKL$^{(1-180)}$-gyrase fusion protein were treated using protocol 1, 2, or 3, as described in **Figure 3A**. At the conclusion of the experiment, cells were analysed for PI uptake using flow cytometry, with a minimum of 5000 cells counted. Data represent three independent experiments, with the exception of the *RIPK3*$^{-/-}$ protocol 3 data where n = 2. Bars indicate the mean and error bars indicate standard error of the mean. Statistics were calculated in GraphPad Prism8, and p values are shown where $p<0.05$. **(E)** U937 cells of various genotypes were treated with inhibitors alone for 24 h to assess their toxicity, then assessed for PI uptake using flow cytometry, with a minimum of 5000 cells counted. Data represent three independent experiments.

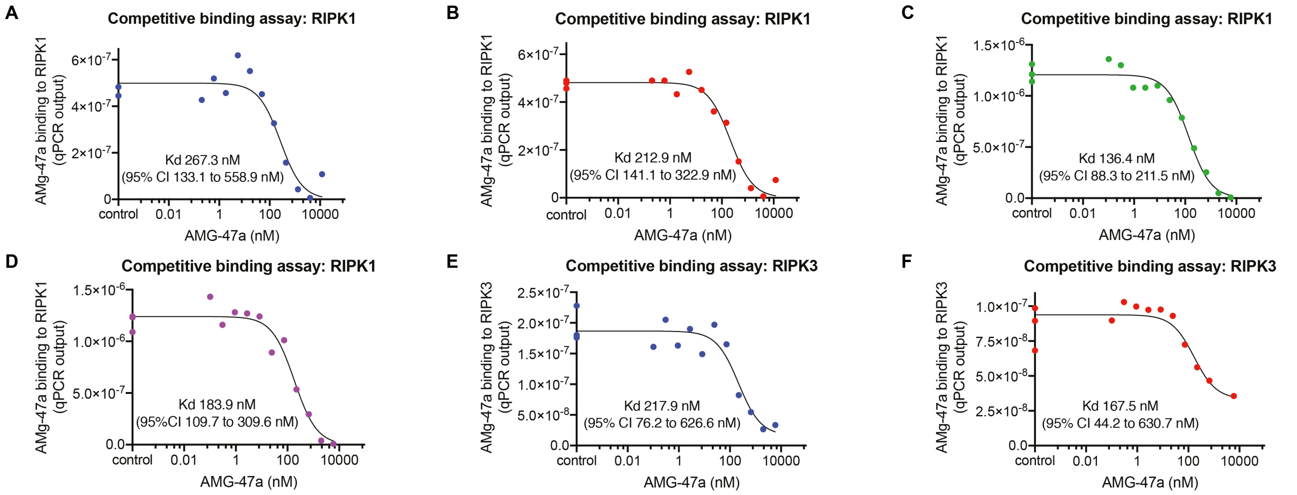

Supplementary Figure 4 – (relates to Figure 6. AMG-47a is a dual RIPK1-RIPK3 inhibitor.) (A-F) AMG-47a binding to RIPK1 (**A-D**) and RIPK3 (**E, F**) was determined in competitive binding assays using the KINOMEscan® Assay Platform (DiscoverX). Graphs were plotted using the raw data supplied from DiscoverX, and a curve was fitted using non-linear regression (GraphPad Prism) to determine the K_d and associated error (95% confidence interval). Each graph represents one individual run of the competitive binding assay, and an analysis of the combined data for each protein can be found in the main text (**Fig. 4F, G**).
