# Supplementary material for: The Lck inhibitor, AMG-47a, blocks necroptosis and implicates RIPK1 in signalling downstream of MLKL
Source: Cell Death Dis. 2022 Apr 1;13(4):291. doi: 10.1038/s41419-022-04740-w (PMC8976052; doi:10.1038/s41419-022-04740-w)
Supplement: Supplementary file 2 — Uncropped western blots [file 41419_2022_4740_MOESM2_ESM.pdf]

Panel 2B

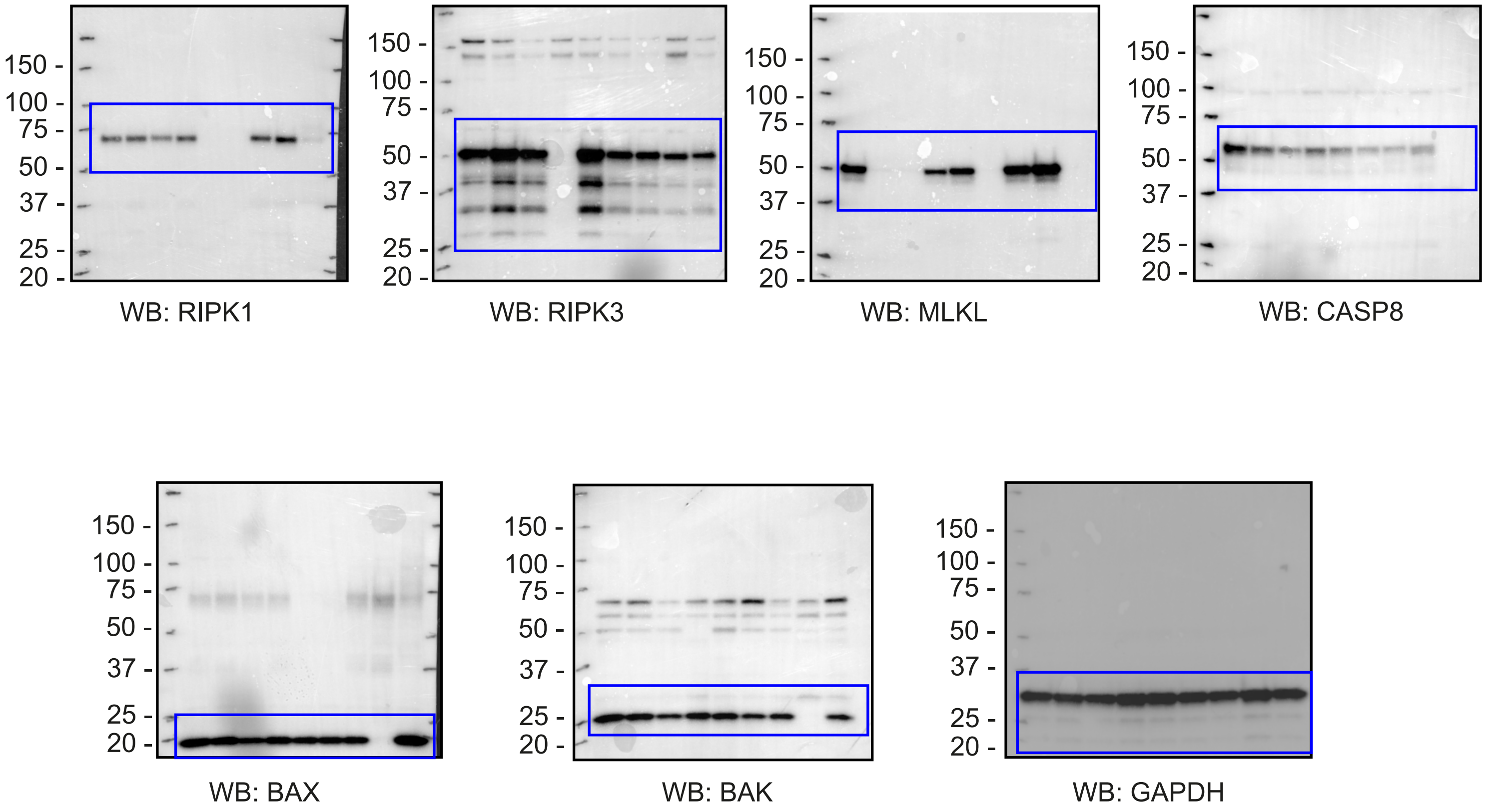

Panel 4C

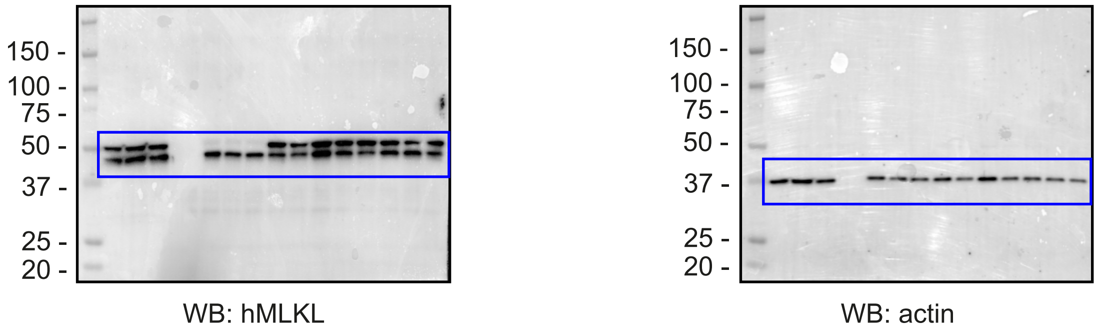

Panel 6A

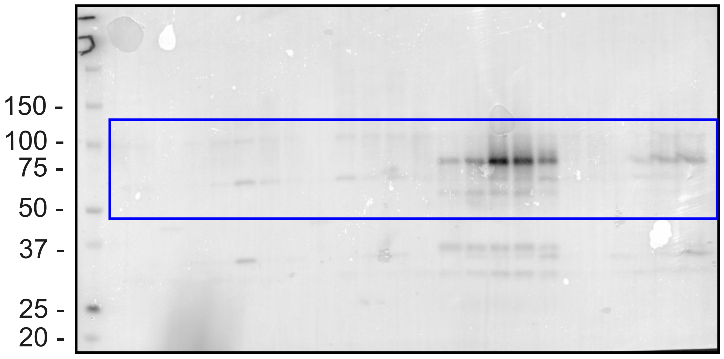

WB: pRIPK1

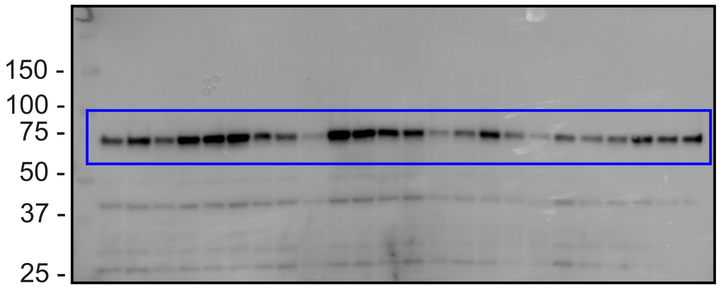

WB: RIPK1

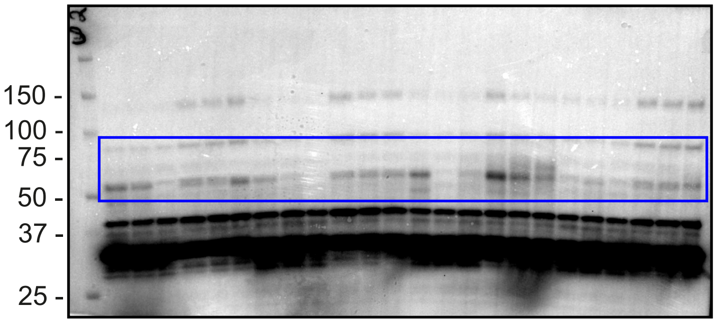

WB: pRIPK3

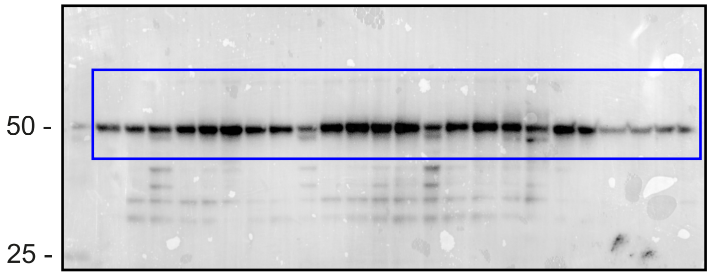

WB: RIPK3

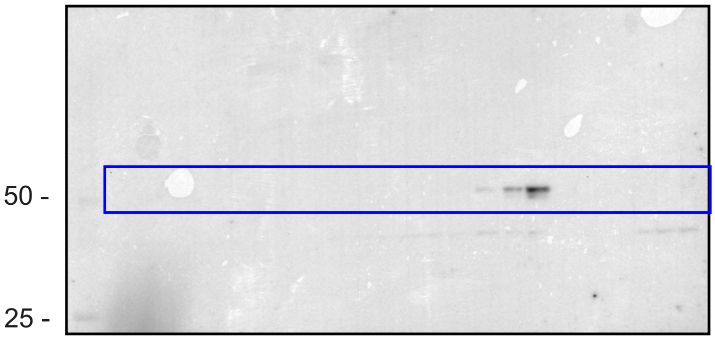

WB: pMLKL

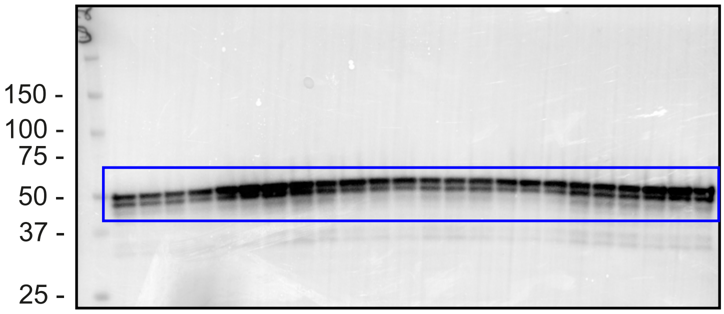

WB: MLKL

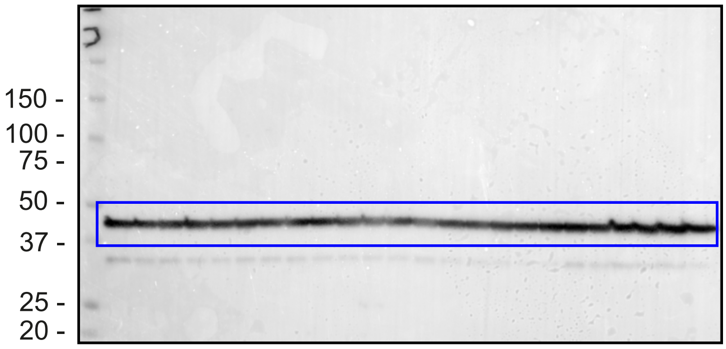

WB: actin

Panel 6B

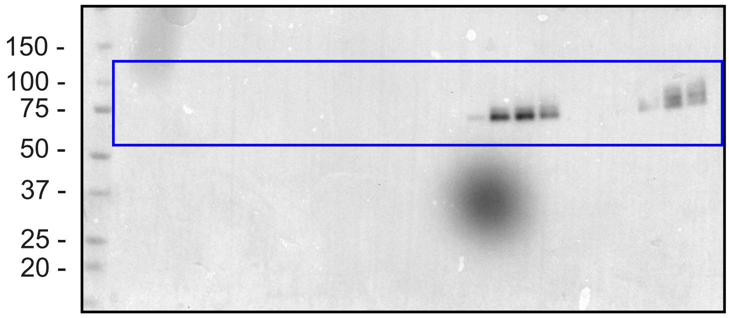

WB: pRIPK1

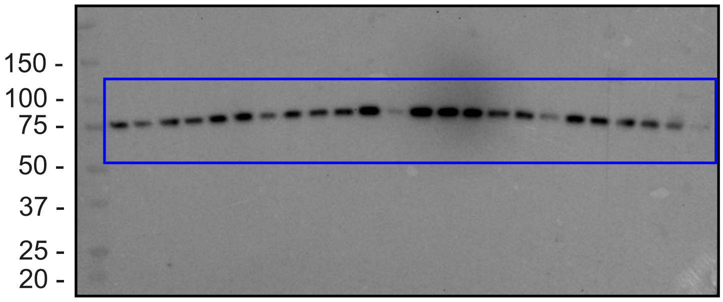

WB: RIPK1

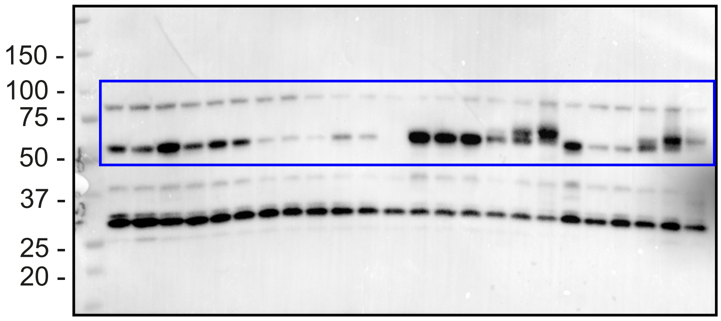

WB: pRIPK3

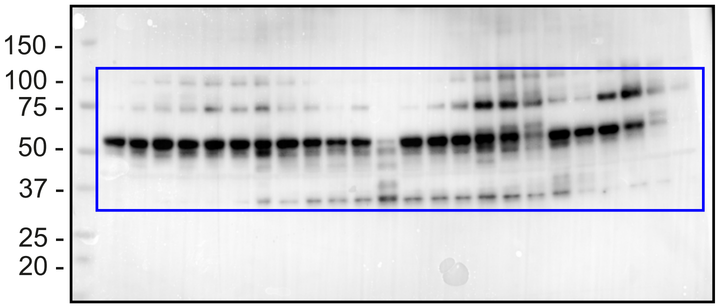

WB: RIPK3

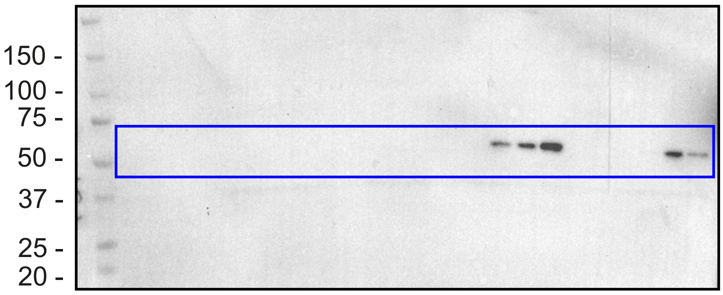

WB: pMLKL

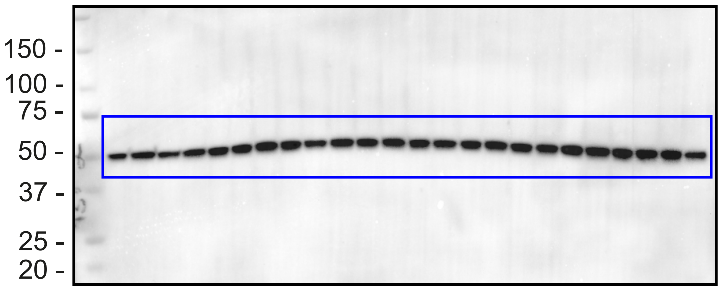

WB: MLKL

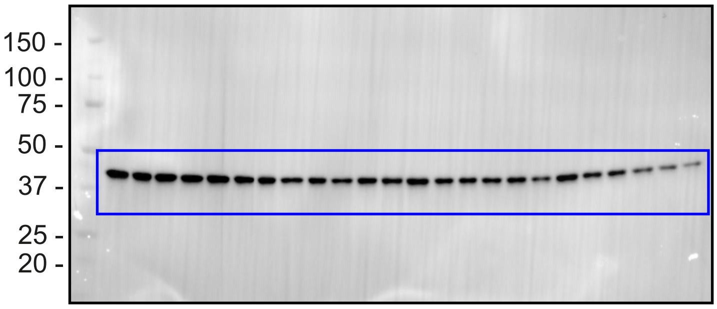

WB: actin

Panel 6C

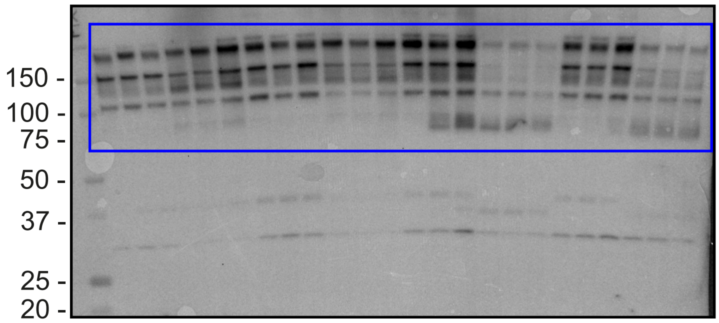

WB: pRIPK1

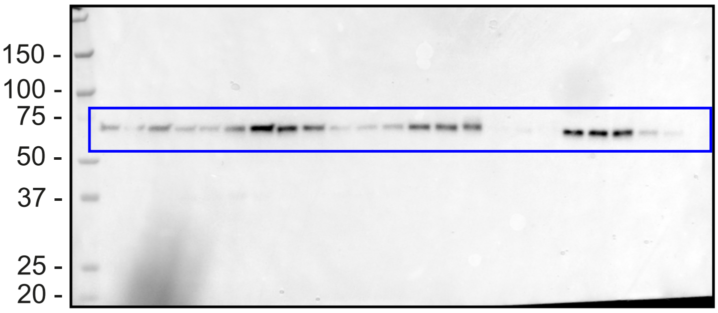

WB: RIPK1

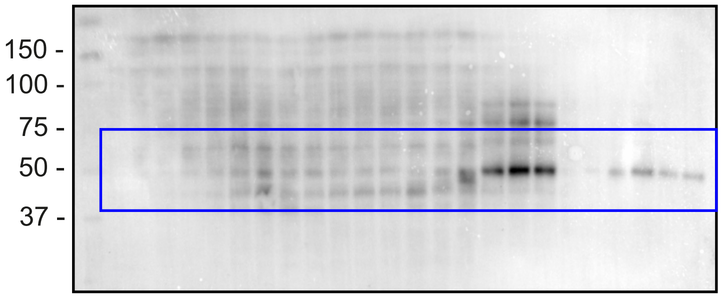

WB: pRIPK3

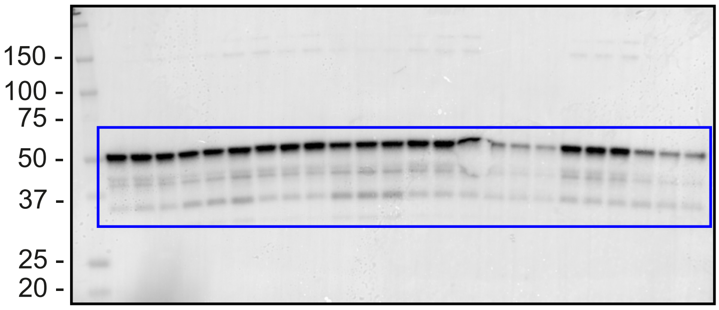

WB: RIPK3

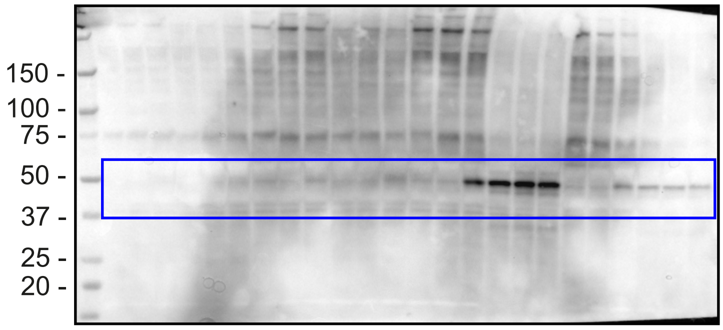

WB: pMLKL

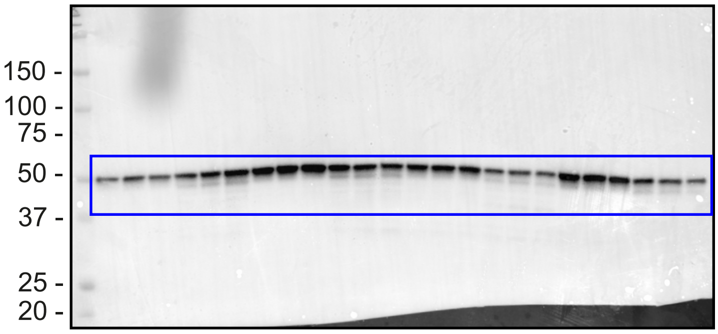

WB: MLKL

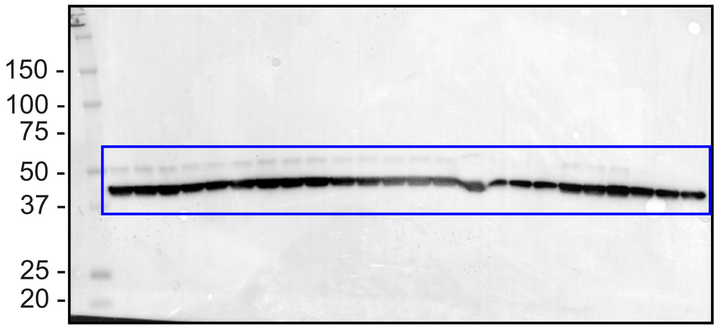

WB: actin

Panel 6D

hRIPK1

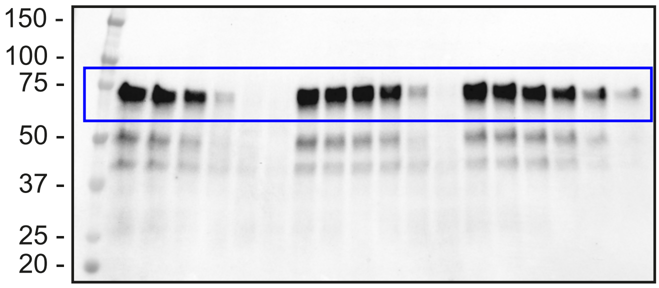

WB: RIPK1

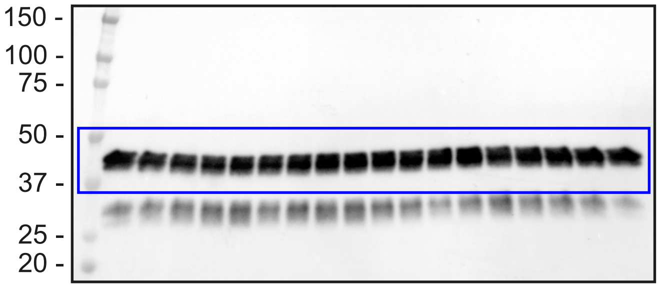

WB: actin

hRIPK3

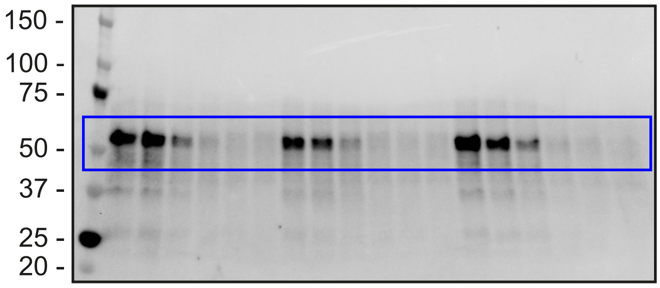

WB: RIPK3

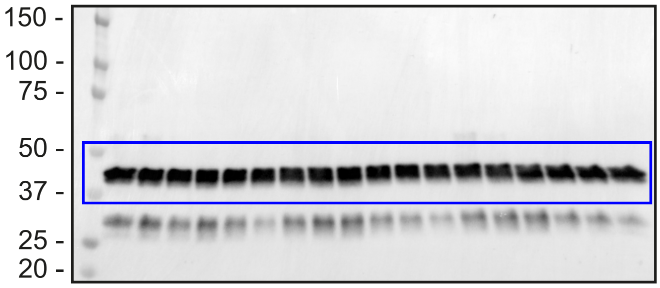

WB: actin

hMLKL

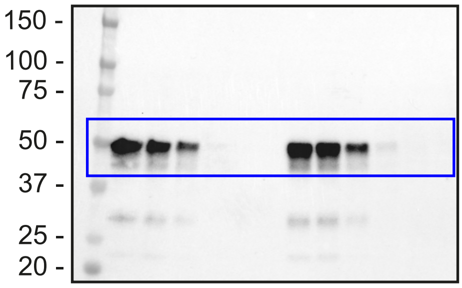

WB: hMLKL

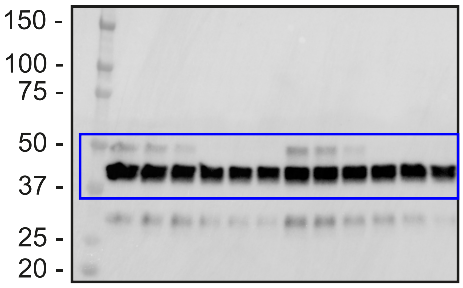

WB: actin

Panel 6E

mRIPK1

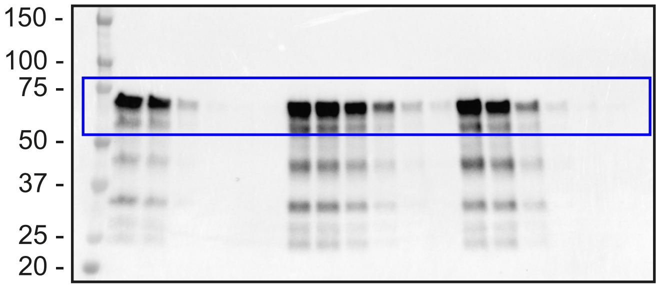

WB: mRIPK1

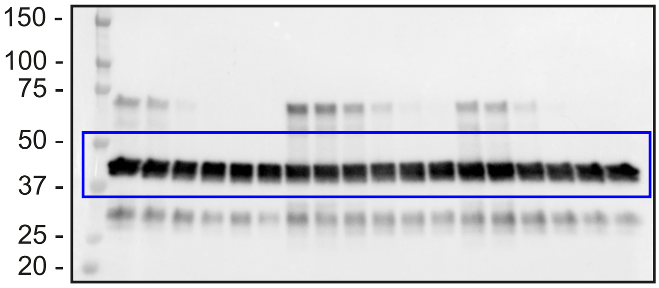

WB: actin

mRIPK3

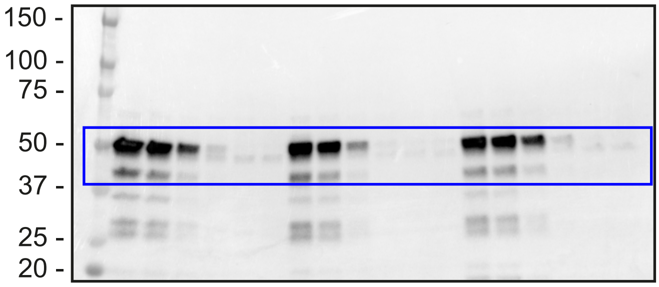

WB: mRIPK3

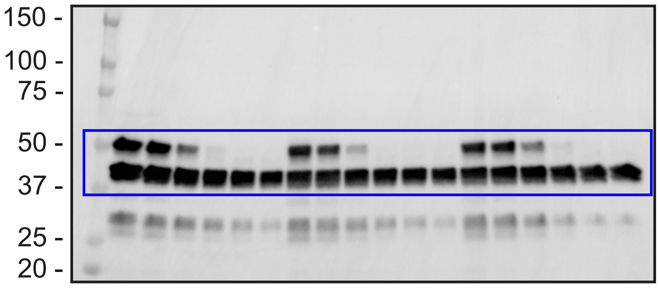

WB: actin

mMLKL

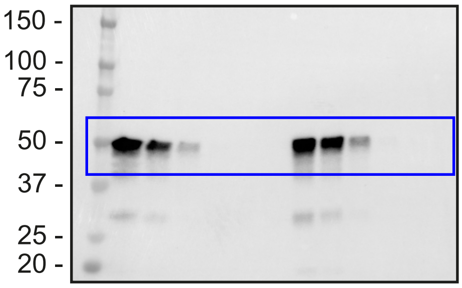

WB: mMLKL

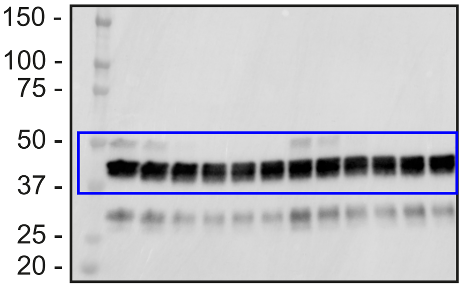

WB: actin

Panel S1E

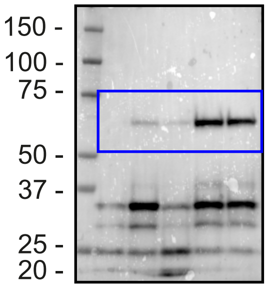

WB: RIPK1

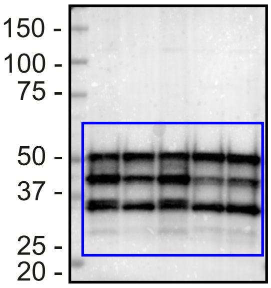

WB: RIPK3

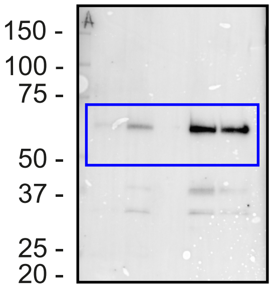

WB: MLKL

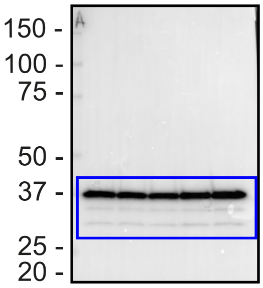

WB:actin
